# Quorum sensing rewires membrane vesicle protein cargo to promote antibiotic persistence in *Streptococcus mutans*

**DOI:** 10.1128/jb.00323-26

**Published:** 2026-07-14

**Authors:** Delphine Dufour, Camila Leiva-Sabadini, Sebastian Aguayo, Céline M. Lévesque

**Affiliations:** 1 Faculty of Dentistry, University of Toronto 70374, Toronto, Ontario, Canada; 2 Institute for Biological and Medical Engineering, Pontificia Universidad Católica de Chile 28033, Santiago, Chile; 3 School of Dentistry, Faculty of Medicine, Pontificia Universidad Católica de Chile 60709, Santiago, Chile; University of Illinois Chicago, Chicago, Illinois, USA

**Keywords:** *Streptococcus mutans*, quorum sensing, peptide signaling, membrane vesicles, persister cells, antibiotic tolerance

## Abstract

Quorum sensing (QS) plays a central role in the adaptive biology of *Streptococcus mutans*, yet the extent to which competence-stimulating peptide (CSP) signaling intersects with membrane vesicle production and function remained unclear. In this study, CSP activation markedly increased membrane vesicle output during the stationary phase and drove extensive changes in vesicle protein composition. Proteomic analysis revealed broad remodeling of vesicle cargo, including shifts in subcellular origin, and enrichment of multiple functional protein classes. Notably, CSP-induced vesicles carried the persistence-associated peptide Pep299, suggesting a link between QS-dependent cargo loading and antibiotic survival. To examine the functional consequences of this remodeling, *S. mutans* cells were pre-exposed to vesicles prior to antibiotic challenge. Vesicles derived from CSP-induced cultures promoted persister formation in a concentration-dependent manner, whereas vesicles from non-induced cultures exhibited minimal activity. Wild-type vesicles increased persistence, and vesicles from Pep299-overexpressing cells further enhanced this phenotype at lower vesicle concentrations. In contrast, vesicles derived from a Δ299 mutant failed to promote persistence, demonstrating that Pep299 is a key determinant of this activity. At higher vesicle abundance, the responses of wild-type and Pep299-enriched vesicles converged, consistent with endogenous CSP-dependent Pep299 loading. Fusion assays using R18 dequenching showed that vesicles from wild-type, Pep299-overexpressing, and Δ299 strains fused with comparable efficiency, indicating that differences in persistence arise from cargo composition rather than altered delivery efficiency. Together, these findings reveal that CSP QS coordinates biogenesis and selective cargo remodeling in *S. mutans* and identify Pep299-containing vesicles as a quorum-regulated mechanism promoting antibiotic persistence.

**IMPORTANCE:**

Quorum sensing enables bacterial populations to coordinate adaptive behaviors, yet its influence on membrane vesicle biology is not fully understood. This study shows that competence-stimulating peptide (CSP) signaling in *Streptococcus mutans* not only increases vesicle production but also remodels vesicle protein cargo, including enrichment of the persistence-associated peptide Pep299. CSP-induced vesicles enhance the formation of persister cells through a cargo-dependent mechanism rather than through changes in vesicle–cell fusion. These findings uncover a previously unrecognized connection between quorum sensing and vesicle-mediated persistence and reveal a strategy through which *S. mutans* modulates community behavior and resilience within the oral biofilm environment.

## INTRODUCTION

Quorum sensing (QS) is a cell–cell signaling mechanism used by bacteria to coordinate gene expression at the population level. This process involves the production, secretion, detection, and response to extracellular signaling molecules that accumulate in the environment as cell density increases (1, 2). QS was first discovered in marine bacteria and linked to population density-dependent coordination of group behaviors (3). As bacterial populations increase, signaling molecules accumulate to a threshold concentration sensed by cognate receptors, initiating coordinated changes in gene expression at the population level (4, 5). Through this mechanism, bacteria regulate a wide range of collective behaviors, including biofilm formation, virulence factor production, bioluminescence, sporulation, and competence development (6–8).

Subsequent work has established that QS is tightly interconnected with bacterial stress response mechanisms, enabling populations to coordinate adaptive behaviors in fluctuating and hostile environments (9). In many species, this relationship is bidirectional: environmental stresses modulate the activity of QS systems, while intact QS pathways are required for survival under adverse conditions and the development of adaptive tolerance responses. In *Streptococcus mutans*, a well-established model organism for oral streptococci, the competence-stimulating peptide (CSP)–ComDE QS is now recognized as a central regulator of general stress responses and population survival strategies rather than a pathway dedicated solely to genetic competence (10–12). CSP signaling is induced under multiple environmental conditions relevant to the oral cavity, including acidic, oxidative, and nutrient-limiting stresses (10). Activation of the ComDE regulatory cascade results in large-scale transcriptional reprogramming that coordinates diverse phenotypic outcomes, enabling population-wide adaptation to environmental challenges (12, 13).

Over the last two decades, extracellular membrane vesicles (MVs) have been recognized as a conserved mechanism of secretion and intercellular interaction in bacteria (14, 15). In gram-positive bacteria, including *S. mutans*, MVs originate from localized extrusion of the cytoplasmic membrane followed by passage through the cell wall at sites of controlled remodeling (16, 17). These nanoscale lipid bilayer structures carry a diverse and selective cargo of biomolecules, including proteins, lipids, nucleic acids, metabolites, and signaling molecules, and function as protected vehicles for extracellular delivery (18, 19). MVs have been implicated in a broad range of biological processes, including intercellular communication, biofilm formation, host interaction, nutrient acquisition, horizontal gene transfer, and antibiotic defense (20, 21). Importantly, vesicle biogenesis and cargo composition are highly regulated processes shaped by the physiological state of the cell and environmental conditions rather than passive byproducts of growth (22, 23). Environmental stresses, including antibiotic exposure, have been shown to markedly influence both vesicle production and protein cargo composition in gram-positive bacteria, resulting in vesicles that can directly modulate antibiotic activity and promote bacterial survival (24, 25).

One important stress-adaptive phenotype regulated by the CSP–ComDE QS system in *S. mutans* is the formation of antibiotic persister cells (26, 27). Persisters are transient phenotypic variants that exhibit high tolerance to bactericidal antibiotics despite genetic susceptibility, typically representing a small subpopulation within an isogenic culture (28, 29). While persisters can arise stochastically, their formation is most frequently triggered by environmental stresses and involves active physiological remodeling, including metabolic reprogramming and suppression of antibiotic target activity (30). The reversible nature of this state contributes significantly to chronic and relapsing infections (31). Previous work from our group established a direct link between environmental stress detection and triggered persister formation under the control of the CSP–ComDE regulatory system in *S. mutans* (26). Through genetic analyses, we identified the gene *pep299* as a key determinant of the triggered persistence phenotype (12, 32). Expression of *pep299* is specifically induced under DNA-damaging conditions and in triggered persister populations, and *pep299* was shown to be both necessary and sufficient for persister formation under these conditions. Notably, *pep299* expression gives rise to an extracellular activity capable of promoting persister formation in neighboring cells, indicative of a population-level signaling function. Bioinformatic analysis of the predicted Pep299 protein revealed several features consistent with extracellular deployment. Pep299 is a small peptide predicted to be processed from a precursor containing an N-terminal leader sequence with a double-glycine motif, a hallmark of peptides destined for secretion in gram-positive bacteria (33). Such features are common among ribosomally synthesized peptides involved in intercellular signaling and stress adaptation. Despite these predictions, the pathway by which the Pep299 peptide is exported and delivered to recipient cells remained unresolved, as no dedicated secretion route could be identified.

In the present study, we investigated the impact of the CSP–ComDE QS system on vesicle biogenesis and protein cargo composition. Given that CSP–ComDE functions as a central stress-response regulatory system in *S. mutans*, and that MVs represent stress-responsive, selective delivery platforms in bacteria, we hypothesized that QS activation intrinsically remodels MV production and cargo. Importantly, because prior work identified *pep299* as a key determinant of triggered persistence and demonstrated an extracellular activity for its predicted gene product without identifying a delivery mechanism, we further postulated that MVs could serve as a vehicle for QS-regulated persistence factors. To test these hypotheses, we combined quantitative proteomic analyses of MVs produced following CSP induction with functional assays assessing the impact of QS-stimulated vesicles on persister cell formation.

## MATERIALS AND METHODS

### Bacterial strains and growth conditions

*S. mutans* UA159 (ATCC 700610) was used as the wild-type (WT) strain. An overexpression strain, referred to as Pep299+, was constructed by introducing the *pep299* gene into the shuttle vector pIB166 and transferring it into an ∆299 background, as previously described (32). Overexpression of *pep299* in the Pep299+ strain was confirmed by RT-qPCR. All strains were grown in Todd-Hewitt broth (BD Difco) supplemented with 0.3% (wt/vol) yeast extract (THYE) at 37°C with 5% CO_2_. Cultures of the Pep299+ strain were maintained in medium supplemented with 10 µg/mL chloramphenicol. Artificial saliva used for surface coating was prepared using the following components: 0.16% (wt/vol) sodium chloride, 0.01% (wt/vol) ammonium nitrate, 0.02% (wt/vol) potassium chloride, 0.03% (wt/vol) potassium citrate, 0.002% (wt/vol) uric acid sodium salt, 0.02% (wt/vol) urea, 0.05% (wt/vol) lactic acid sodium salt, and 0.3% (wt/vol) mucin type II. The solution was autoclaved for 20 min at 121°C and cooled before use.

### Membrane vesicle isolation and characterization

Membrane vesicles (MVs) were isolated from logarithmic- and stationary-phase cultures as previously described (34), with minor modifications. Overnight cultures (20 h) were diluted 1:100 into 100 mL fresh THYE broth, with or without 50 ng/mL synthetic CSP (SGSLSTFFRLFNRSFTQA; Shanghai Royobiotech Co. Ltd), a concentration previously shown to induce antibiotic persistence in *S. mutans* (12, 32), and incubated at 37°C until reaching the desired growth phase. Cells were removed by centrifugation at 4,000 × *g* for 15 min at 4°C, and the resulting supernatants were passed through a 0.22 µm membrane filter (Thermo Scientific Nalgene) to remove residual bacteria. Sterile culture supernatants were then concentrated using 100 kDa ultrafiltration units (Millipore Sigma). Concentrated supernatants were subjected to ultracentrifugation at 140,000 × *g* for 1 h at 4°C using an Optima L-70 ultracentrifuge (Beckman Coulter). MV pellets were resuspended in 500 µL sterile, filtered phosphate-buffered saline (PBS). Aliquots of each MV preparation were plated on THYE agar and incubated for 24 h at 37°C to confirm sterility. MV concentration and size distribution were measured by nanoparticle tracking analysis (NTA) using a NanoSight 300 tracker (Malvern) at the Structural & Biophysical Core Facility, The Hospital for Sick Children (Toronto, Canada). MV protein content was quantified using the QuantiPro BCA assay kit (Millipore Sigma). MV preparations were stored at −80°C until further use.

### MV preparation for proteomic analysis

MVs resuspended in PBS were mixed with six volumes of precooled (−20°C) acetone (HPLC Plus, ≥99.9%, Millipore Sigma) and incubated overnight at −20°C. Protein pellets were collected by centrifugation at 10,000 × *g* for 10 min at 4°C, washed once with 100 µL cold acetone, and resuspended in 100 µL of 25 mM ammonium bicarbonate (pH 7.8; ReagentPlus, ≥99.9%, Millipore Sigma). Disulfide bonds were reduced by adding 1 mM dithiothreitol and incubating the samples for 1 h at 56°C. Reduced cysteine residues were alkylated by adding 1 mM iodoacetamide and incubating for 1 h at room temperature in the dark. Proteins were digested overnight at 37°C with sequencing-grade trypsin (Promega) at a final concentration of 12.5 ng/µL. Digestion was stopped by adding formic acid to a final concentration of 1% (vol/vol).

### LC-MS/MS analysis of MV proteins

Tryptic peptides were purified using C18 spin columns (Pierce, Thermo Scientific) and dried by vacuum centrifugation, resuspended in 0.1% (vol/vol) formic acid, and analyzed on a Q Exactive Plus LC-MS/MS system (Thermo Fisher Scientific). Peptide separation was performed on a gravity-packed analytical column (75 µm internal diameter, 10 cm length; New Objective) containing 100 Å, 5 µm Magic C18AQ particles (Michrom). Mobile phase A consisted of 0.1% (vol/vol) formic acid, and mobile phase B consisted of 0.1% (vol/vol) formic acid in 80% (vol/vol) acetonitrile. Chromatography was carried out at a flow rate of 300 nL/min using the following gradient: 0–5 min, 4–10% B; 5–53 min, 10–40% B; 53–62 min, 40–95% B; 62–80 min, 95–100% B; followed by a 10-min hold at 100% B. Peptides were introduced into the Q Exactive Plus mass spectrometer using an EASY nLC 1200 nano-LC system (Thermo Fisher Scientific) equipped with a Nano Flex-ESI source (35). Mass data were acquired in data-dependent acquisition mode, with automatic switching between one full scan and up to 10 MS/MS scans. Full MS spectra were acquired at a resolution of 70,000 (at *m/z* 400) with an automatic gain control (AGC) target of 1 × 10^6^ and a maximum injection time of 120 ms. MS/MS spectra were acquired at a resolution of 17,500 (at *m/z* 400) with an AGC target of 1 × 10^6^, a maximum injection time of 120 ms, a first mass cutoff of 140 *m/z*, and a normalized collision energy of 27. Unassigned precursor ions and ions with charge states of 1 or >5 were excluded from MS/MS selection. Dynamic exclusion was set to 20 s to minimize repeated sequencing of identical precursor ions.

### Proteomic data processing and protein identification

Raw MS/MS data were acquired using Xcalibur 4.2 (Thermo Fisher Scientific) and analyzed with Proteome Discoverer 2.4 (Thermo Fisher Scientific) using a label-free quantification workflow. Spectra were searched against a database containing the complete *S. mutans* UA159 proteome (Taxon identifier 210007; UniProt proteome ID UP000002512). Database search included a precursor mass tolerance of 10 ppm, a fragment mass tolerance of 0.02 Da, and trypsin/P specificity, allowing up to two missed cleavages. Carbamidomethylation of cysteine was set as a fixed modification, while methionine oxidation and protein N-terminal acetylation were included as variable modifications. Peptide and protein identifications were filtered to a false discovery rate (FDR) of 1%. Proteins were accepted only if supported by at least two peptides. Peptide abundance was quantified based on peptide peak area measurements. Protein abundance was then calculated from the abundances of the peptides assigned to each protein according to the label-free quantification workflow in Proteome Discoverer 2.4.

### Persister cell assay

Overnight cultures of the WT strain were diluted 1:100 into 300 µL of fresh THYE broth in 96-well polystyrene microtiter plates pre-coated with artificial saliva. MVs isolated from CSP-induced stationary-phase cultures were added to final concentrations of 0, 50, 100, or 200 µg/mL. After incubation for 2 h at 37°C, cultures were treated with 10× MIC of ofloxacin (20 µg/mL) for 24 h at 37°C to eliminate non-persister cells (26). Samples collected immediately prior to antibiotic addition (*T*_0_) and after 24 h (*T*_24_) were washed once with PBS, serially diluted, and plated onto THYE agar. Colony-forming units (CFU) were enumerated after 48 h of incubation at 37°C. The percentage of persister cells was calculated as follows: (CFU count at *T*_24_/CFU count at *T*_0_) × 100.

### Membrane vesicle−cell fusion assay

Fusion between MVs and *S. mutans* cells was quantified using an R18 (octadecyl rhodamine B chloride) fluorescence dequenching assay (36). For labeling, 100 µg of MVs were resuspended in 1 mL of 0.1 M NaCl/0.05 M Na_2_CO_3_ (pH 9.2) and incubated with 40 µg/mL R18 (Invitrogen) for 1 h at room temperature in the dark. Labeled vesicles were collected by ultracentrifugation (140,000 × *g*, 4°C, 1 h) and washed twice with filtered PBS. The final MV pellet was resuspended in 750 µL filtered PBS supplemented with one complete EDTA-free Protease Inhibitor Cocktail tablet (Millipore Sigma). Fusion reactions were set up by mixing 50 µL of R18-labeled MVs with 250 µL of PBS-washed overnight *S. mutans* cells in dark 96-well microtiter plates. A control containing labeled MVs mixed with PBS alone was included to monitor spontaneous dequenching. Fluorescence was monitored over time using *λ*_ex_ = 560 nm and *λ*_em_ = 595 nm. Fusion was terminated by adding 0.3% (vol/vol) Triton X-100, which fully dequenches the probe and generates maximum fluorescence. Fusion efficiency was calculated as percent fluorescence dequenching (%FD) using:


$$\%FD=\left[\frac{F-F_{i}}{F_{\max}-F_{i}}\right]\times 100$$

where *F* is fluorescence at a given time point, *F_i_* is the initial fluorescence of labeled MVs before cell addition, and *F*_max_ is the fluorescence measured after Triton X-100 treatment (37).

### Statistical analysis

All assays were performed with at least three independent biological replicates. Data are presented as means ± standard deviations. Statistical analyses were carried out using GraphPad Prism version 9.0 (GraphPad Software). Comparisons between two groups were performed using an unpaired Student’s *t*-test, while multiple group comparisons were analyzed using one-way analysis of variance (ANOVA) followed by Tukey’s multiple-comparison test. Differences were considered statistically significant at *P* < 0.05.

## RESULTS

### CSP signaling enhances membrane vesicle production

**Fig 1:**
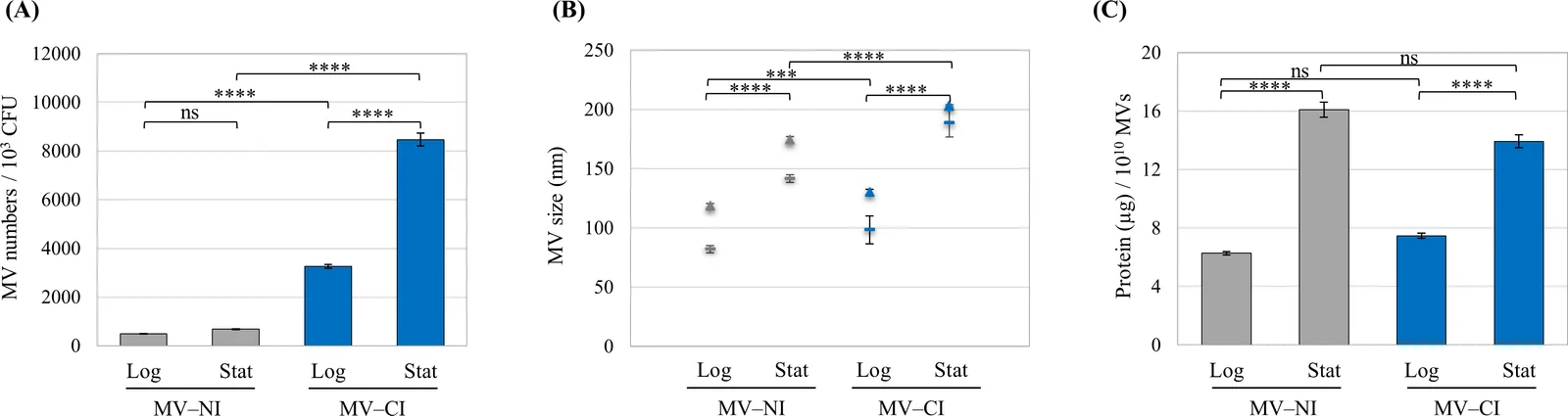
MV production by *S. mutans* WT under non-induced and CSP-induced conditions. MV from non-induced (MV–NI) and CSP-induced (MV–CI) cultures were isolated at logarithmic (Log) and stationary (Stat) growth phases. (**A**) MV production yield expressed as the number of MVs per 10^3^ CFU present in cultures at the time of MV isolation. (**B**) MV size distributions (nm), with mean diameters shown as triangles and mode values indicated as horizontal bars. (**C**) MV protein content (µg) normalized to 10^10^ MVs. Data represent means ± standard deviations from three biological replicates. Statistical significance was assessed using one-way ANOVA followed by Tukey’s multiple-comparison test. Significance levels are indicated as follows: ns, not significant; ***, *P* < 0.001; and ****, *P* < 0.0001.

To determine whether QS influences MV biogenesis in *S. mutans*, we quantified MV production in cultures grown in the absence or presence of CSP. MVs were isolated from clarified culture supernatants collected during the logarithmic and stationary growth phases. Vesicle preparations derived from non-induced cultures were designated MV–NI, whereas those obtained under CSP induction were designated MV–CI. To account for differences in growth phase cell densities, MV yields were normalized to CFU present at the time of MV isolation. CSP stimulation markedly increased MV production at both growth phases. During logarithmic growth, CSP exposure resulted in approximately a sevenfold increase in MV yield compared with MV–NI preparations. This effect was even more pronounced at stationary phase, where CSP induction generated an approximately 12-fold increase in vesicle output (Fig. 1A).

CSP induction also altered vesicle size profiles in a growth phase-dependent manner. During logarithmic growth, MV–CI displayed a significantly larger average diameter than MV–NI (130.5 ± 2.1  nm vs 118.7 ± 2.0  nm, respectively) (Fig. 1B). In contrast, CSP had an even more pronounced effect at the stationary phase, where MV–CI exhibited significantly larger vesicles (203.0 ± 1.0  nm) compared with MV–NI (174.6 ± 2.7  nm) (Fig. 1B). Notably, MV–CI collected at the stationary phase also showed the most homogeneous vesicle size distribution, with a mean diameter of 203.0 ± 1.0  nm and a mode of 188.7 ± 11.8  nm. Representative NanoSight particle size distribution outputs for stationary-phase MV–NI and MV–CI samples are provided in Fig. S1.

To determine whether these changes in MV abundance and size reflected altered cargo loading, protein content was quantified and normalized to vesicle number. A pronounced growth phase effect was observed for both MV–NI and MV–CI, with stationary-phase vesicles containing more than twice the amount of protein per 10^10^ MVs compared with their logarithmic-phase counterparts (Fig. 1C). Specifically, MV–NI increased from ~6 µg at log phase to ~16 µg at stationary phase, while MV–CI increased from ~7.5 to ~14 µg. Despite these substantial differences across growth phases, protein loading was comparable between MV–NI and MV–CI within each phase, indicating that CSP induction does not significantly impact the amount of protein packaged per vesicle. Instead, CSP primarily influences MV abundance and size rather than altering total protein cargo per vesicle.

### CSP induction drives extensive remodeling of MV protein cargo

**Fig 2:**
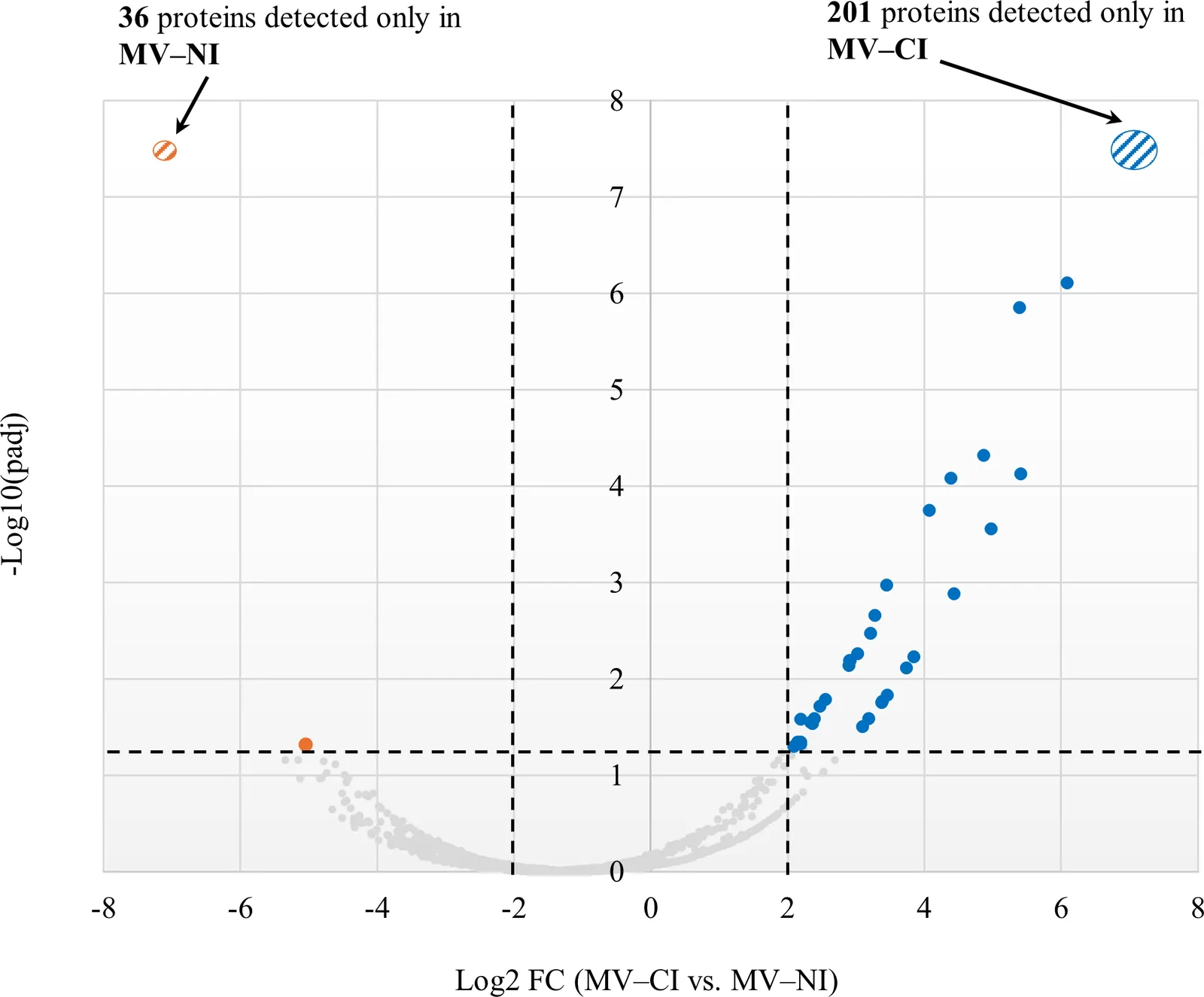
Differential abundance of MV proteins under CSP-induced and non-induced conditions. Volcano plot showing proteins differentially abundant between MV–CI and MV–NI isolated at stationary phase. The *x*-axis represents the log_2_ fold-change (MV–CI vs MV–NI), and the *y*-axis shows −log_10_(adjusted *P*). Vertical dashed lines indicate the significance thresholds of log_2_FC ≥ 2 or ≤−2, and the horizontal dashed line denotes an adjusted *P* value of 0.05. Proteins detected only in MV–NI (36 proteins), and therefore depleted in MV–CI, are shown as hatched orange symbols, whereas proteins detected only in MV–CI (201 proteins) are shown as hatched blue symbols. Proteins significantly enriched in MV–CI appear as solid blue points on the right side of the plot, and the single protein significantly enriched in MV–NI appears as a solid orange point on the left side of the plot. Gray points represent proteins that did not meet significance criteria.

Having established that CSP induction increases MV production and modifies vesicle size, we next examined whether these CSP-dependent effects extend to changes in MV protein cargo. To this end, a label-free quantitative proteomic analysis was performed to compare the composition of MV–NI and MV–CI isolated at stationary phase. Across all samples, a total of 1,680 proteins were identified. Applying a significance threshold of log₂FC ≥2 or ≤−2 with an adjusted *P* value < 0.05, 270 proteins displayed differential abundance between MV–CI and MV–NI (Fig. 2). Of these 270 proteins, the majority (233 proteins, 86.3%) were enriched in MV–CI. This enriched group consisted primarily of proteins unique to MV–CI (201 proteins; 74.4% of all differentially abundant proteins), along with 32 proteins shared by both vesicle types but present at significantly higher levels in MV–CI (Table S1). In contrast, only 37 proteins (13.7%) were depleted in MV–CI, consisting almost entirely of proteins unique to MV–NI (36 proteins; 13.3%), plus a single protein present at higher abundance in MV–NI than MV–CI (Table S2). Together, these results reveal a substantial CSP-dependent rewiring of MV protein cargo, characterized by a strong bias toward proteins enriched or selectively packaged in MV–CI. Notably, the 201 proteins uniquely detected in MV–CI represent approximately 10% of the annotated protein-coding capacity of the UA159 genome, indicating robust and selective cargo recruitment into vesicles produced under CSP-induced conditions.

### CSP induction alters the subcellular origins of MV protein cargo

**Fig 3:**
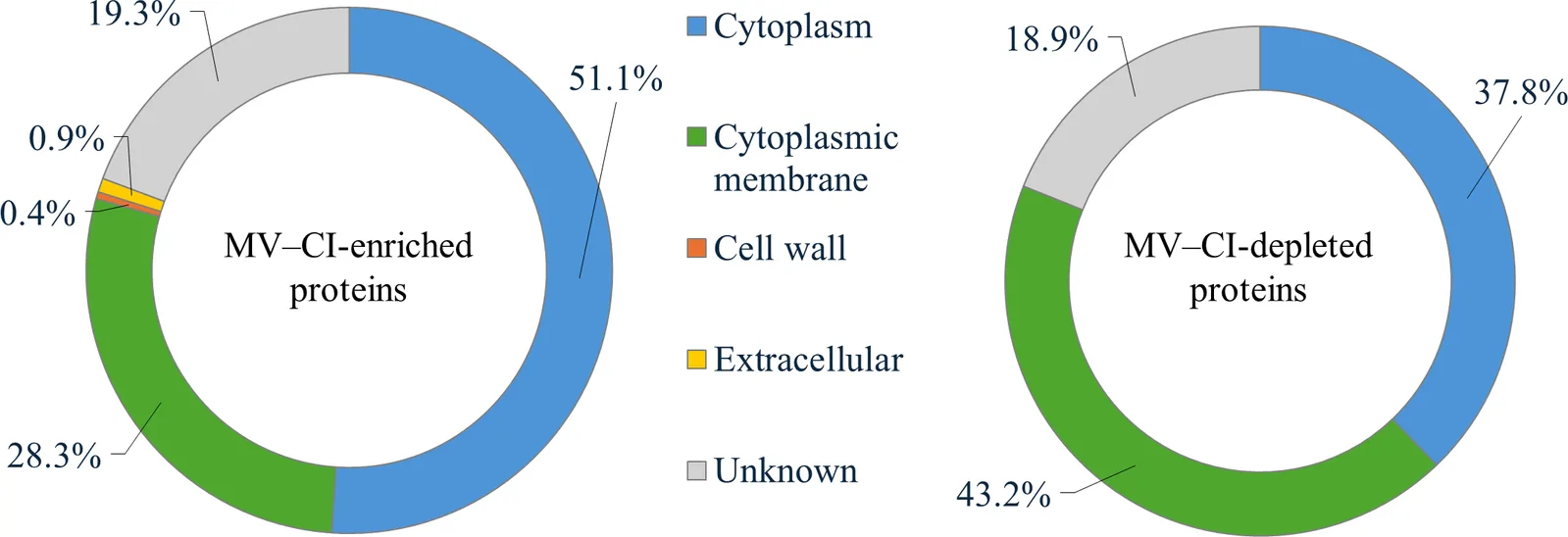
Predicted subcellular localization of MV–CI-enriched and MV–CI-depleted proteins. The PSORTb 3.0 algorithm was used to predict the subcellular localization of MV–CI-enriched proteins (left) and MV–CI-depleted proteins (right). Donut plots show the proportion of proteins assigned to cytoplasm, cytoplasmic membrane, cell wall, extracellular space, or unknown localization. MV–CI-depleted proteins include only cytoplasmic, cytoplasmic membrane, and unknown categories.

To determine whether the extensive CSP-dependent changes in MV protein abundance were accompanied by shifts in the cellular origins of MV cargo, we analyzed the predicted subcellular localization of all differentially abundant proteins using PSORTb 3.0 (38) (Fig. 3). Among MV–CI-enriched proteins, the largest fraction was predicted to be cytoplasmic (51.1%), followed by cytoplasmic membrane proteins (28.3%). Smaller proportions corresponded to cell wall-associated (0.9%) and extracellular proteins (0.4%), while 19.3% lacked a predicted localization. In contrast, MV–CI-depleted proteins displayed a simpler distribution: the majority were predicted to be either cytoplasmic membrane proteins (43.2%) or cytoplasmic proteins (37.8%), with the remaining 18.9% classified as of unknown localization. Notably, no cell wall or extracellular proteins were detected in the MV–CI-depleted group.

Although the differences between enriched and depleted proteins are not dramatic, they reveal a modest shift in the types of proteins incorporated into MVs under CSP-induced conditions. Cytoplasmic proteins form the largest fraction of MV–CI-enriched cargo, whereas MV–CI-depleted proteins are relatively more membrane-associated. These patterns complement the broader CSP-dependent proteomic changes and point to subtle remodeling of MV cargo selection during QS activation.

### CSP induction enriches MV cargo for specific functional protein classes

**Fig 4:**
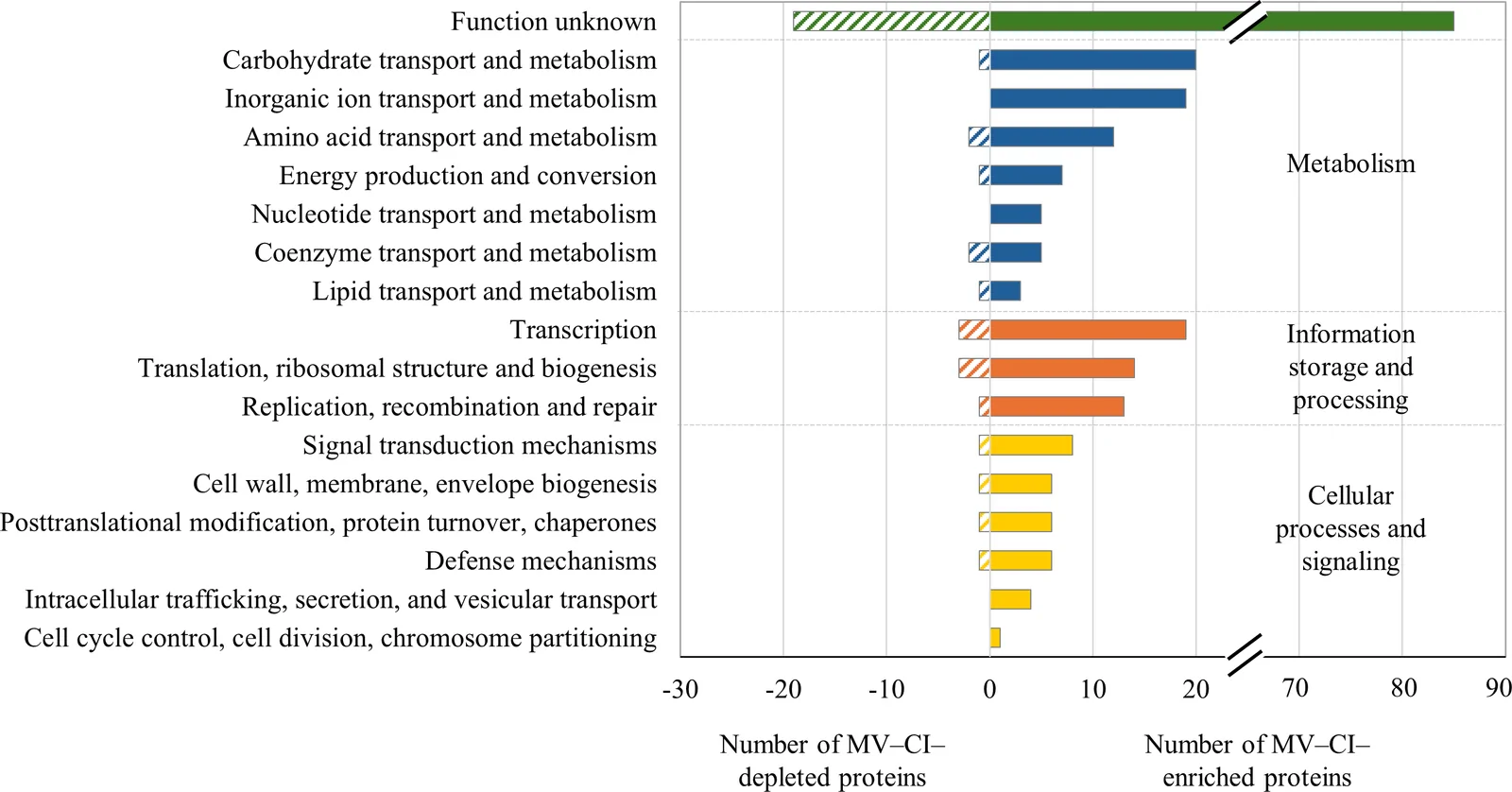
COG functional classification of MV–CI-enriched and MV–CI-depleted proteins. Proteins differentially abundant between MV–CI and MV–NI were assigned to COG functional categories, which group proteins based on predicted biological roles. Bars indicate the number of proteins enriched in MV–CI (right, solid colors) or depleted in MV–CI (left, hatched colors) within each COG category. The “defense mechanisms” category includes proteins associated with stress responses and protective functions, such as antimicrobial resistance and detoxification systems. Proteins classified as “function unknown” correspond to those lacking functional annotation.

To characterize the functional landscape of proteins differentially incorporated into MVs under CSP-induced conditions, we classified MV–CI-enriched and MV–CI-depleted proteins using Clusters of Orthologous Groups (COG) categories (39) (Fig. 4). Among MV–CI-enriched proteins, 148 were assigned to known COG categories. Nearly half (47.3%) belonged to metabolic functions; 31.1% were associated with information storage and processing; and 21.6% mapped to cellular processes and signaling, including categories related to stress adaptation such as defense mechanisms. In addition, 85 enriched proteins were categorized as function unknown and are included in Fig. 4. MV–CI-depleted proteins showed a more restricted distribution, with most assigned to metabolic pathways or information-processing categories, and only a small proportion associated with cellular process-related functions. An additional 19 depleted proteins were classified as function unknown (Fig. 4).

**Fig 5:**
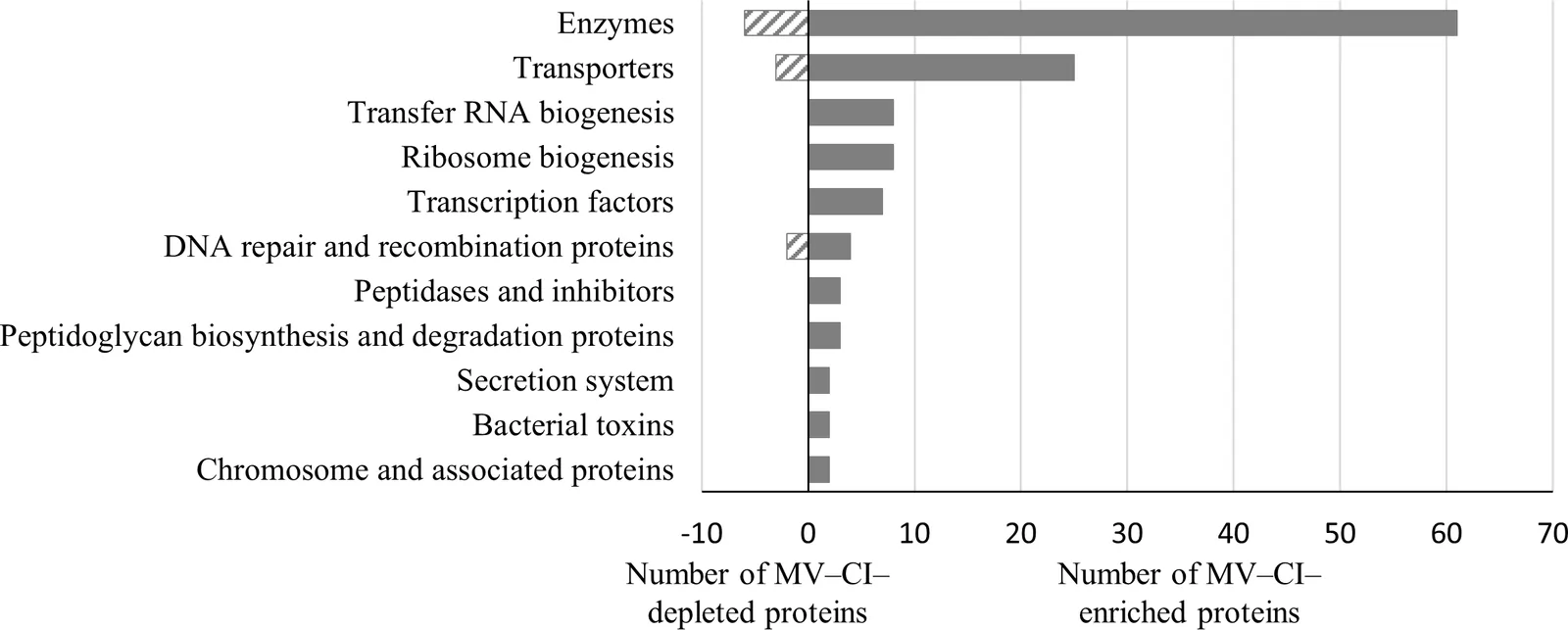
KEGG BRITE hierarchical functional categories of MV–CI-enriched and MV–CI-depleted proteins. KEGG BRITE annotations were used to classify the functional attributes of proteins differentially abundant between enriched and depleted fractions. Only BRITE categories represented by more than one protein are shown. MV–CI-enriched proteins are displayed as solid bars (right), and MV–CI-depleted proteins are displayed as hatched bars (left).

To further explore functional characteristics, we analyzed KEGG BRITE hierarchical categories (40) (Fig. 5). Only BRITE categories represented by more than one protein were included in the figure for clarity. Consistent with the COG analysis, MV–CI-enriched proteins were dominated by enzymes, which comprised more than half of all BRITE-classified enriched proteins, along with a large group of transporters. Additional enriched categories included tRNA, ribosome biogenesis factors, and transcription factors. In contrast, MV–CI-depleted proteins were almost exclusively classified as enzymes or transporters, with minimal representation in other BRITE categories.

Together, the COG and KEGG BRITE analyses show that CSP induction not only alters the abundance and subcellular origin of MV cargo but also broadens its functional repertoire. CSP-induced vesicles contain a more diverse set of proteins spanning metabolic pathways, macromolecular synthesis, stress responses, and cell-envelope–associated processes, consistent with the extensive proteomic remodeling triggered by QS activation in *S. mutans*.

### CSP-induced membrane vesicles selectively enrich stress-defense proteins and the persister factor Pep299

CSP-induced MVs displayed a distinct proteomic profile enriched in multiple classes of stress-response proteins (Table 1). Notably, several oxidative and redox defense proteins were uniquely associated with MV–CI, including the peroxidase resistance protein Dpr, the thioredoxin reductase TrxB, the thiol peroxidase Tpx, and the methionine sulfoxide reductase MsrA, all of which play essential roles in neutralizing reactive oxygen species and repairing oxidative damage (41). Their selective packaging indicates that CSP signaling promotes the export of proteins that help *S. mutans* cope with oxidative stress.

**TABLE 1:** Stress defense mechanism-related proteins unique to MV–CI

Category	Gene ID	UniProt accession	Protein function
Oxidative and redox stress defense	SMU.540	Q8DVF0	Peroxidase resistance protein
	SMU.869	Q8DUN5	Thioredoxin reductase
	SMU.924	Q8DUK3	Thiol peroxidase
	SMU.1622	Q8DSY4	Methionine sulfoxide reductase
pH, osmotic and ion-homeostasis stress	SMU.328	Q8DVY1	Carbonic anhydrase
	SMU.1013	Q8DUC7	Mg^2+^/citrate symporter
	SMU.1057	Q8DU87	Acid tolerance operon, SatE
	SMU.1061	P96468	Acid tolerance operon, YlxM
	SMU.1289	Q8DTP4	Permease, chloride channel
	SMU.1693	I6L8Z1	Magnesium protection factor
	SMU.2119	Q8DRU5	Osmoprotectant ABC transporter, permease
DNA repair and genome maintenance	SMU.1215	Q8DTV8	Uracil DNA glycosylase
	SMU.1870	Q8DSD1	DNA mismatch repair protein
	SMU.2087	Q8DRX1	3-methyl-adenine DNA glycosylase
Cell envelope stress and global regulation	SMU.337	Q8DVX3	Membrane protein insertase
	SMU.485	Q8DVK0	Membrane protein LiaF
Interspecies competition and antimicrobial activity	SMU.151	Q8DWB6	Mutacin IV, NlmB
	SMU.656	Q8DV52	Lantibiotic transport system permease, MutE2
	SMU.1146	Q8DU13	Nukacin resistance, response regulator
	SMU.2080	Q8DRX6	Bacteriocin transcriptional regulator, BrsR
	SMU.2109	I6L919	Multidrug efflux pump
Persister-related factors	SMU.299	Q8CVC8	Bacteriocin-like peptide, Pep299
	SMU.896	Q8DUM3	mRNA-degrading endonuclease

MV–CI carried a comprehensive set of proteins linked to acid, osmotic, and ion-homeostasis stresses, all found exclusively in the CSP-induced condition. These included the carbonic anhydrase SMU.328, which contributes to cytoplasmic pH regulation; the Mg²^+^/citrate symporter CitM, likely involved in citrate uptake and ionic balance (42); and two *sat* operon components, SatE and YlxM, known mediators of acid tolerance (43). MV–CI also contained the chloride channel permease SMU.1289, which may enhance ionic equilibrium (44), and the osmoprotectant ABC transporter OpuBB, a key importer of compatible solutes during hyperosmotic stress (45). MV–CI further carried SMU.1693, previously annotated as a putative hemolysin but now recognized as the *S. mutans* homolog of MpfA, a magnesium efflux pump essential for Mg²^+^-dependent stress tolerance (46). Collectively, this set of stress-associated proteins underscores the role of MV–CI in equipping *S. mutans* to withstand the extreme and fluctuating physicochemical conditions characteristic of the oral biofilm.

The vesicles also contained an exclusive set of proteins involved in DNA repair and genome maintenance, including the uracil DNA glycosylase SMU.1215, the mismatch repair protein MutS2, and the 3-methyl-adenine DNA glycosylase SMU.2087. Their presence suggests that MV–CI may contribute to stress adaptation by delivering DNA repair-associated proteins to recipient cells, potentially enhancing genome maintenance at the population level during CSP-activated stress responses.

Cell envelope-associated proteins were another prominent feature of MV–CI, with YidC1 (SMU.337) and LiaF both detected exclusively in the induced vesicles. These proteins are collectively tied to membrane protein insertion and envelope integrity monitoring (47, 48), aligning with the idea that CSP-driven vesiculation is tightly synchronized with cell envelope remodeling and maintenance pathways.

Moreover, MV–CI were enriched in antimicrobial and competitive fitness factors, including the mutacin IV precursor NlmB, the lantibiotic transport permease MutE2, the nukacin-associated response regulator LcrR, the bacteriocin transcriptional regulator BrsR, and a multidrug efflux pump (SMU.2109). These bacteriocin-related systems are well recognized as key ecological determinants that enhance microbial competitiveness and shape interspecies interactions within polymicrobial communities, particularly in the dental biofilm (49, 50).

Finally, two persister-associated proteins were uniquely observed in MV–CI: a RelE-family mRNA-degrading toxin (26) and, critically, Pep299, a bacteriocin-like peptide previously implicated in promoting persister cell formation in *S. mutans* (32). The selective inclusion of Pep299 in MV–CI is particularly noteworthy, as it suggests that CSP-induced vesiculation may represent a mechanism through which *S. mutans* mobilizes persistence-associated factors. This observation reinforces the connection between QS and stress tolerance while providing a mechanistic basis to investigate whether MV–CI are functionally capable of promoting persister formation and to further define the contribution of Pep299 to this phenotype.

### CSP-induced MVs promote persister formation

**Fig 6:**
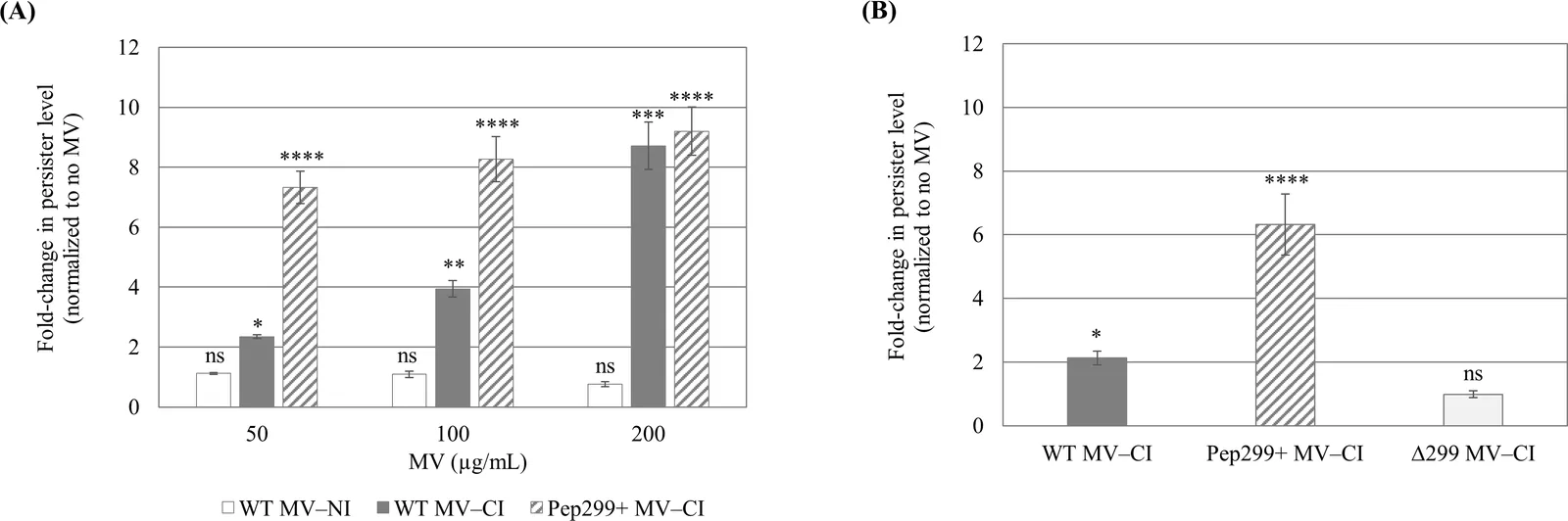
Impact of CSP-induced MVs on persister formation. *S. mutans* cells were pre-exposed to MVs and subsequently treated with 10× MIC of ofloxacin for 24 h. Persister levels were quantified by CFU enumeration and expressed as fold-change relative to the no-MV control. (**A**) Dose-dependent effects of MVs isolated from WT CSP-induced (WT MV–CI), WT non-induced (WT MV–NI), and Pep299-overexpressing CSP-induced (Pep299+ MV–CI) cultures. (**B**) Persister levels following treatment with 50 µg/mL MVs isolated from WT (WT MV–CI), Pep299-overexpressing (Pep299+ MV–CI), or Pep299-deficient (Δ299 MV–CI) strains. Data represent means ± standard deviations from three biological replicates. Statistical significance was determined using an unpaired Student’s *t*-test (GraphPad Prism 9.0): ns, not significant; *, *P* < 0.05; **, *P* < 0.01; ***, *P* < 0.001; and ****, *P* < 0.0001.

Having established that CSP signaling dramatically enhances MV production at stationary phase and alters their protein cargo, we next evaluated the ability of these MVs to promote persister formation. *S. mutans* WT cells were pre-exposed to increasing concentrations of MVs isolated from stationary-phase cultures grown either in the absence or presence of CSP, followed by challenge with 10× MIC of ofloxacin. MVs derived from CSP-induced cultures exhibited a strong dose-dependent increase in persister levels, yielding approximately twofold, fourfold, and ninefold increases at 50, 100, and 200 µg/mL, respectively, relative to the no-MV control (Fig. 6A). In contrast, MVs isolated from non-induced cultures showed little to no effect across all concentrations tested (Fig. 6A). These results indicate that CSP signaling confers persister-promoting activity to MVs.

We next investigated whether this CSP-dependent effect could be attributed, at least in part, to enrichment of the persister-associated peptide Pep299. To this end, we compared MV–CI derived from the WT and the Pep299+ strain, a *pep299* overexpression strain constructed in the ∆299 background (32). RT-qPCR analysis confirmed that *pep299* expression was increased by ~10-fold (10.24 ± 0.28) in the Pep299+ strain relative to the WT, validating this strain as a model for Pep299 enrichment. Consistent with this interpretation, Pep299+ MV–CI generated substantially higher persister levels than WT MV–CI at lower concentrations (Fig. 6A). At 50 µg/mL, Pep299+ MV–CI produced approximately threefold higher persister levels than WT MV–CI, whereas at 100 µg/mL, this enhancement remained evident at approximately twofold. At 200 µg/mL, however, persister levels converged between WT and Pep299+ MVs (Fig. 6A). This plateau effect is consistent with WT MV–CI already carrying substantial amounts of Pep299, thereby limiting the impact of additional Pep299 enrichment.

To directly assess the contribution of Pep299 to the persistence phenotype, we next compared MVs derived from CSP-induced cultures of WT, ∆299, and Pep299+ strains (Fig. 6B). WT MV–CI increased persister levels by approximately twofold relative to the no-MV control. In contrast, Pep299+ MV–CI induced a markedly stronger response, resulting in an approximately sixfold increase in persister formation. Notably, MVs derived from the ∆299 mutant failed to promote persistence and remained comparable to the no-MV control.

**Fig 7:**
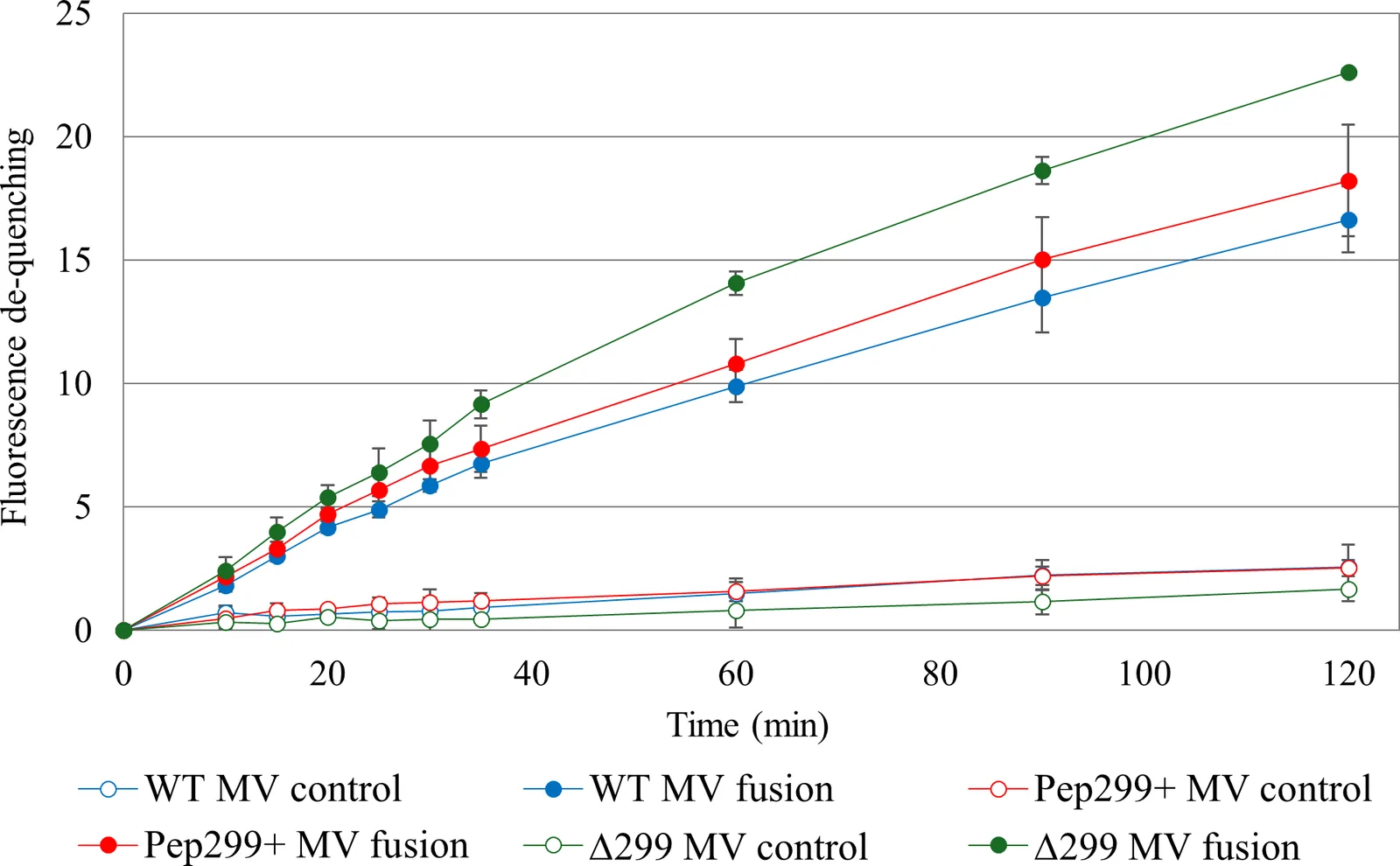
Fusion kinetics of WT, Pep299+, and ∆299 MVs measured by R18 dequenching. Fluorescence dequenching was monitored over time to assess fusion of WT MV–CI, Pep299+ MV–CI, and ∆299 MV–CI with *S. mutans* cells. Fusion curves (solid symbols) were compared with dye-only controls (open symbols). All MV types exhibited robust, time-dependent fusion, with no significant differences observed among WT, Pep299+, and ∆299 MVs. Data represent means ± standard deviations from three independent biological replicates.

To determine whether the differential persister-promoting activity of MV–CI could be attributed to differences in vesicle–cell delivery rather than cargo composition, we assessed MV–cell fusion using an R18 dequenching assay. MVs derived from WT, Pep299+, and ∆299 strains all exhibited robust, time-dependent fusion with *S. mutans* cells, with fluorescence signals clearly exceeding those of dye-only controls (Fig. 7). Notably, all three MV types displayed broadly comparable fusion kinetics, indicating that their interaction with recipient cells occurs with similar efficiency. Although minor differences in signal intensity were observed, these did not correlate with the ability of MVs to promote persister formation, as ∆299 MVs retained strong fusion activity despite lacking persister-inducing effects. Thus, the enhanced persister formation associated with Pep299+ MVs and the loss of activity in ∆299 MVs are attributable to differences in vesicle cargo, specifically Pep299 enrichment, and not from altered delivery efficiency.

Collectively, these results demonstrate that CSP-induced vesicles are sufficient to promote persister formation and that this activity is strongly dependent on the presence of Pep299. Enrichment of Pep299 further enhances this survival phenotype, particularly at lower MV concentrations, whereas deletion of *pep299* abolishes the persister-promoting effect of MVs. These findings position Pep299 as a major determinant of the persistence-inducing activity of MV–CI and support a model in which QS-driven peptide cargo loading modulates the survival properties of *S. mutans* vesicles.

## DISCUSSION

Bacterial extracellular MVs constitute a heterogeneous population of lipid-enclosed nanoparticles produced throughout growth and biofilm development (14, 18). Once regarded as stochastic byproducts of membrane turnover, MV biogenesis is now recognized as a conserved and regulated feature of bacterial physiology (51). In biofilms, MVs contribute not only to matrix architecture but also function as active mediators of intercellular communication, nutrient exchange, horizontal gene transfer, and stress adaptation (16, 20, 24, 52). Importantly, MV cargo encapsulation is increasingly understood to be selective rather than random, enabling bacteria to tailor vesicle content in response to specific environmental and physiological cues (53, 54).

In this study, we demonstrate that activation of the CSP QS pathway in *S. mutans* actively rewires MV biogenesis and cargo composition to promote antibiotic persistence. While previous studies have reported increased MV production in response to antibiotic stress, such changes are often interpreted as indirect consequences of membrane damage or detoxification (55). By inducing QS in the absence of antibiotics, our data show that CSP signaling alone is sufficient to drive robust vesicle production and selective packaging of stress- and persistence-associated cargo. These findings position MVs as downstream effectors of QS-regulated persistence programs and provide a direct mechanistic link between QS activation, vesicle cargo selection, and community-level antibiotic tolerance.

Consistent with MV biogenesis being a regulated response, CSP signaling markedly enhanced MV production in *S. mutans* in a growth phase-dependent manner. CSP stimulation increased MV yield approximately seven-fold during mid-log growth and twelve-fold at stationary phase, indicating that QS activation alone can promote robust vesicle biogenesis. CSP also altered vesicle size distributions, generating subpopulations of enlarged vesicles while maintaining a constant protein content per vesicle. Stationary phase MV–CI exhibited the greatest size homogeneity, suggesting that QS coordinates vesicle production, maturation, or release when cell density and stress are maximal. The absence of increased protein loading per vesicle argues against nonspecific membrane shedding and instead supports a regulated biogenesis process.

Previous proteomic analyses have demonstrated that *S. mutans*-derived MVs can carry enzymes and even complete, active metabolic pathways, enabling biological functions to occur external to the bacterial cell (34, 56). Notably, MV cargo remodeling observed here following CSP stimulation closely parallels patterns previously reported for MVs derived from *S. mutans* biofilms, which were enriched in proteins associated with oxidative stress defense, redox homeostasis, metabolic adaptation, and envelope stress responses (34). Together with the pronounced quantitative and physical remodeling of MVs observed in response to CSP signaling, these findings suggest that QS not only regulates vesicle production but also programs vesicle functionality in ways that recapitulate key features of the biofilm state. Proteomics analysis revealed that MV–CI are selectively enriched in proteins associated with oxidative and redox stress defense, pH and ionic homeostasis, DNA repair, cell envelope stress responses, antimicrobial resistance, and persistence. The coordinated enrichment of these functional categories supports a model in which MV–CI represent organized extracellular stress-adaptive units rather than inert byproducts of membrane dynamics. MV–CI uniquely contained multiple redox-active enzymes involved in ROS detoxification and repair of oxidatively damaged proteins, consistent with an expanded role for CSP-mediated QS in promoting community-level oxidative stress resilience. Likewise, the selective packaging of proteins linked to pH regulation, osmotic balance, and ion homeostasis closely aligns with the aciduric lifestyle of *S. mutans* and supports a role for MVs in reinforcing stress tolerance under conditions of high cell density and environmental acidification. The enrichment of proteins involved in DNA repair and genome maintenance further suggests that QS anticipates genotoxic stress and biases vesicle cargo toward protective functions associated with persistence and survival in dense, stressed bacterial communities. Although CSP signaling is known to regulate competence-associated pathways, the extent to which vesicle cargo selection is linked to competence-specific regulation remains unclear and warrants future investigation.

The most mechanistically informative finding was the identification of Pep299 peptide exclusively in MV–CI. This observation is particularly significant in light of our previous work demonstrating that *pep299* is a QS-activated gene that plays a central role in antibiotic persister formation in *S. mutans* (32). In that study, *pep299* was shown to be essential for stress-induced persistence, actively secreted, and capable of inducing persister formation in neighboring cells in co-culture (32). Expression of *pep299* was also induced under DNA damage conditions and linked to regulation of a QS-inducible toxin, positioning *pep299* at the core of QS-triggered persistence physiology. The present study extends these findings by identifying MVs as a functional delivery platform for Pep299 peptide. We show that MV–CI are not only enriched in Pep299 but are themselves sufficient to promote persister formation. Pre-exposure of *S. mutans* cells to stationary phase MV–CI increased antibiotic persister levels in a clear dose-dependent manner. Moreover, vesicles derived from Pep299-overexpressing strain exhibited significantly enhanced persister-promoting activity at lower MV concentrations compared with WT vesicles, whereas MVs derived from ∆299 mutant failed to induce persistence. Importantly, WT, Pep299+, and ∆299 MVs fused with recipient cells with comparable efficiency, indicating that the observed differences in persister formation arise from variation in vesicle cargo rather than altered vesicle uptake or membrane fusion. These results provide direct functional evidence that Pep299 is a major determinant of the persistence-inducing capacity of MV–CI, with its enrichment further amplifying this activity under limiting conditions. At higher MV concentrations, persister levels induced by WT and Pep299+ MVs converged, consistent with MV–CI already containing substantial amounts of Pep299 at elevated MV yields. This plateau effect supports a threshold-based model in which Pep299 acts as a major determinant of vesicle-mediated persistence, such that maximal induction is achieved once a sufficient quantity of peptide is delivered to recipient cells. Importantly, these findings indicate that while Pep299 is a key contributor to MV-mediated persistence, it may not be the sole determinant, consistent with the presence of complementary stress-defense and persistence-associated cargo within MV–CI.

Our data further indicate that the biological activity of Pep299 depends critically on its mode of delivery. Although Pep299 promotes persistence when genetically expressed or vesicle-associated, the synthetic peptide alone failed to induce persister formation under standard assay conditions (32). This finding argues against a simple diffusion-based or receptor-mediated signaling mechanism and instead supports a model in which Pep299 requires vesicle-mediated transport to exert its function. Encapsulation within MVs likely facilitates intracellular delivery via membrane fusion, overcoming permeability barriers that limit the activity of the free peptide. Vesicle association may also enhance peptide stability by protecting Pep299 from proteolytic degradation and dilution in the extracellular milieu. Support for this vesicle-dependent delivery model comes from recent work on the highly hydrophobic thiopeptide bacteriocin micrococcin P1 (MP1) produced by *Staphylococcus aureus*, a gram-positive bacterium (57). In that system, antimicrobial activity was strictly dependent on incorporation into MVs. MV-associated MP1 was present at high local concentrations and potently active against target cells, whereas the free peptide exhibited minimal activity due to poor solubility in aqueous environments. Importantly, this study provided direct evidence that bacterial MVs can fuse with the cytoplasmic membrane of gram-positive bacteria to deliver peptide cargo to intracellular targets. Together, these findings establish MVs as a general solution to the challenge of secreting and disseminating hydrophobic bioactive peptides (58, 59). By analogy, Pep299 appears to function as a vesicle-delivered persistence effector rather than a freely diffusible signal, enabling stability, intracellular access, and coordinated dissemination of persistence signals beyond immediate cell–cell contact within structured biofilms.

Collectively, our findings highlight MVs as a distinct and regulated secretion mechanism that contributes directly to antibiotic persistence, providing, to our knowledge, the first functional evidence that MV-mediated cargo delivery promotes persister cell formation in bacteria. In the context of a microbial community, MV release by individual cells may confer protective benefits to neighboring cells of the same species, thereby enhancing population-level survival under stress. Such a mechanism allows coordination of stress responses beyond the limits of direct cell–cell contact or classical diffusion-based signaling. Notably, our preliminary data indicate that Pep299 does not stimulate vesicle biogenesis itself, but is selectively packaged into MV–CI, supporting a model in which MVs primarily serve as vehicles for its release, stabilization, and functional delivery. Together, these results expand the functional repertoire of gram-positive bacterial MVs and position them as central mediators of cooperative stress adaptation and phenotypic heterogeneity in biofilm-associated populations.

## Data Availability

The mass spectrometry proteomics data generated in this study have been deposited in the MassIVE repository (https://massive.ucsd.edu) and are publicly available under accession number MSV000102160.

## SUPPLEMENTAL MATERIAL

Supplemental figure and tables jb.00323-26-s0001.pdf Figure S1, and Tables S1 and S2.

ASM does not own the copyrights to Supplemental Material that may be linked to, or accessed through, an article. The authors have granted ASM a non-exclusive, world-wide license to publish the Supplemental Material files. Please contact the corresponding author directly for reuse.
