# Correction for Dufour et al., “Quorum sensing rewires membrane vesicle protein cargo to promote antibiotic persistence in *Streptococcus mutans*”

**DOI:** 10.1128/jb.00468-26

**Published:** 2026-09-18

**Authors:** Delphine Dufour, Camila Leiva-Sabadini, Sebastian Aguayo, Céline M. Lévesque

## AUTHOR CORRECTION

Volume 208, no. 8, e00323-26, 2026, https://doi.org/10.1128/jb.00323-26. The Acknowledgments section was inadvertently omitted from the published version of the article. The section should read as follows: “The authors gratefully acknowledge the University of Toronto Centre for the Analysis of Genome Evolution and Function (CAGEF) for proteomics support and sincerely thank Dr. Pauline Wang and Inga Kireeva for their expertise and assistance with proteomic analyses. The authors also thank Dr. Zhao for helpful discussions and valuable scientific input.

This work was supported by the Natural Sciences and Engineering Research Council of Canada (NSERC) Discovery Grant (RGPIN-2019-06454) awarded to C.M.L.”

The authors regret this omission.

