# Supplementary material for: Quorum sensing rewires membrane vesicle protein cargo to promote antibiotic persistence in Streptococcus mutans
Source: J Bacteriol. 2026 Jul 14;208(8):e00323-26. doi: 10.1128/jb.00323-26 (PMC13492114; doi:10.1128/jb.00323-26)
Supplement: Supplementary file 1 [file jb.00323-26-s0001.pdf]

SUPPLEMENTAL MATERIAL

Quorum sensing rewires membrane vesicle protein cargo to promote antibiotic persistence in *Streptococcus mutans*

Delphine Dufour,[1] Camila Leiva-Sabadini,[2] Sebastian Aguayo,[2,3] Céline M. Lévesque[1✉]

[1]Faculty of Dentistry, University of Toronto, Toronto, Ontario, Canada
[2]Institute for Biological and Medical Engineering, Pontificia Universidad Católica de Chile, Santiago, Chile
[3]School of Dentistry, Faculty of Medicine, Pontificia Universidad Católica de Chile, Santiago, Chile
✉Corresponding author: celine.levesque@dentistry.utoronto.ca

Fig. S1A: Stationary phase MV–NI

NANOSIGHT

No CSP released MVs 50X 2025-03-28 13-25-45

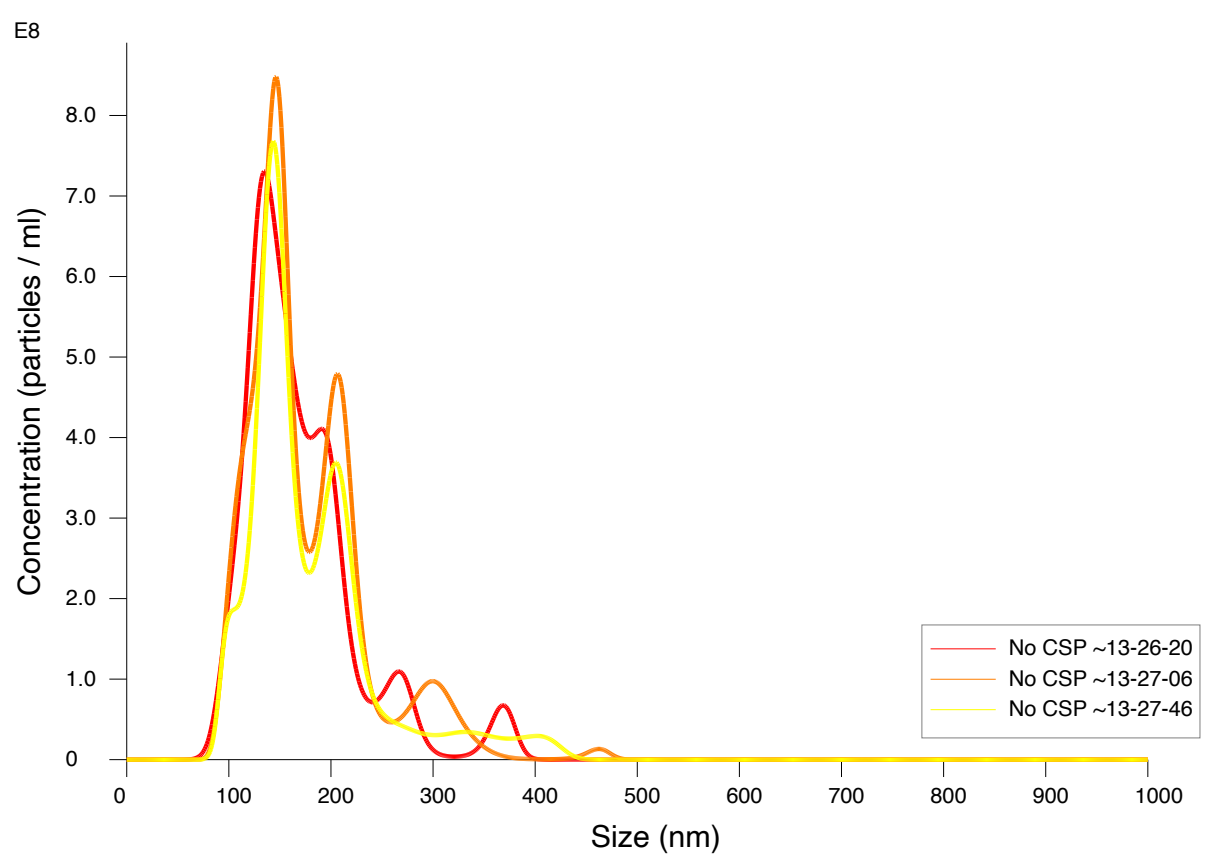

FTLA Concentration / Size graph for Experiment:
No CSP released MVs 50X 2025-03-28 13-25-45

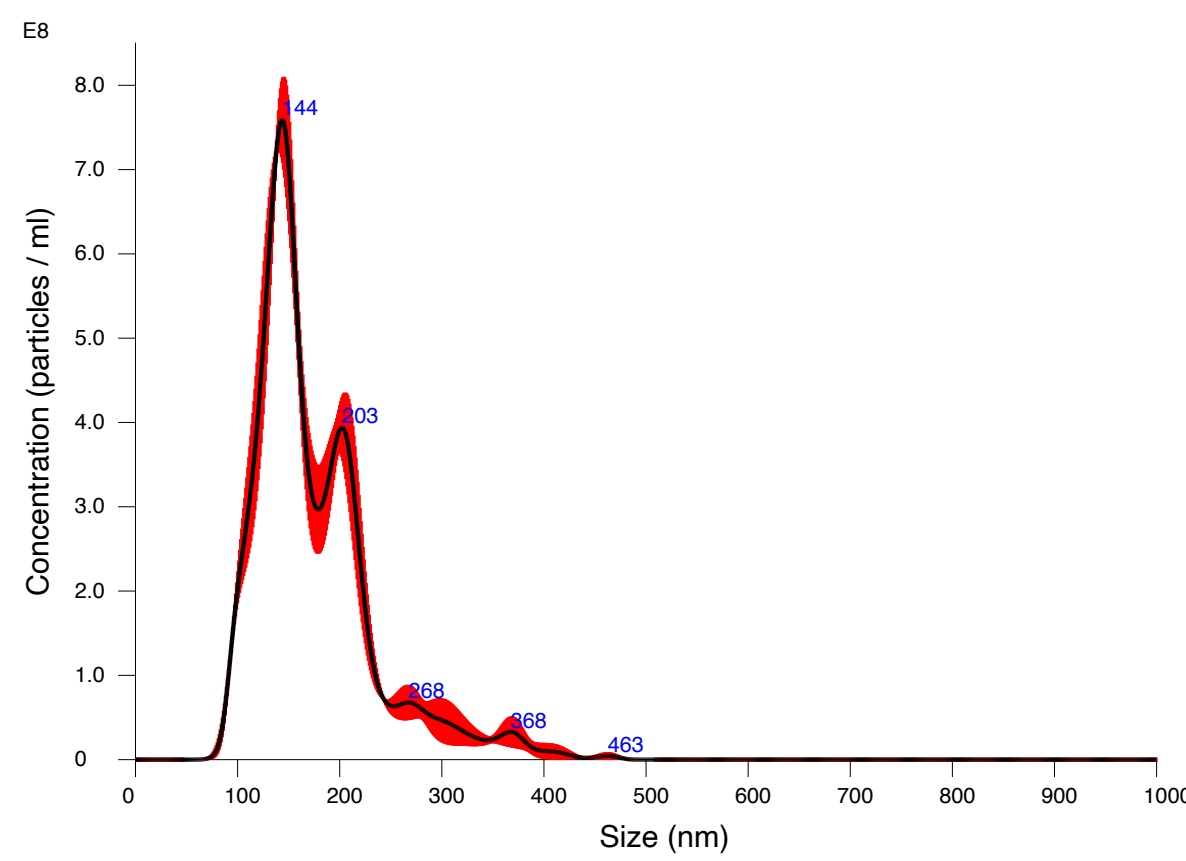

Averaged FTLA Concentration / Size for Experiment:
No CSP released MVs 50X 2025-03-28 13-25-45
Error bars indicate + / -1 standard error of the mean

Included Files

No CSP released MVs 50X 2025-03-28 13-26-20
No CSP released MVs 50X 2025-03-28 13-27-06
No CSP released MVs 50X 2025-03-28 13-27-46

Details

NTA Version:	NTA 3.4 Build 3.4.4
Script Used:	SOP Standard Measurement 11-47-07AM 28Mar2025.txt
Time Captured:	13:25:45 28/03/2025
Operator:	Delphine
Pre-treatment:	
Sample Name:	No CSP released MVs
Diluent:	1/50
Remarks:	

Capture Settings

Camera Type:	sCMOS
Laser Type:	Green
Camera Level:	15
Slider Shutter:	1206
Slider Gain:	245
FPS	25.0
Number of Frames:	749
Temperature:	23.3 ºC
Viscosity:	(Water) 0.922 - 0.923 cP
Dilution factor:	5 x 10e1
Syringe Pump Speed:	15

Analysis Settings

Detect Threshold:	21
Blur Size:	Auto
Max Jump Distance:	Auto: 11.6 - 12.2 pix

Results

Stats: Merged Data

Mean:	174.4 nm
Mode:	143.6 nm
SD:	59.9 nm
D10:	116.0 nm
D50:	157.6 nm
D90:	247.4 nm

Stats: Mean +/- Standard Error

Mean:	174.6 +/- 2.7 nm
Mode:	141.6 +/- 3.4 nm
SD:	59.9 +/- 2.4 nm
D10:	116.5 +/- 1.5 nm
D50:	157.6 +/- 0.7 nm
D90:	247.9 +/- 3.0 nm
Concentration (Upgrade):	6.35e+10 +/- 3.46e+09 particles/ml
	132.0 +/- 4.5 particles/frame
	128.1 +/- 3.8 centres/frame

Concentration measurements may be unreliable
See summary file for more info

Fig. S1B: Stationary phase MV–CI

NANOSIGHT CSP released 500X 2025-03-28 13-39-49

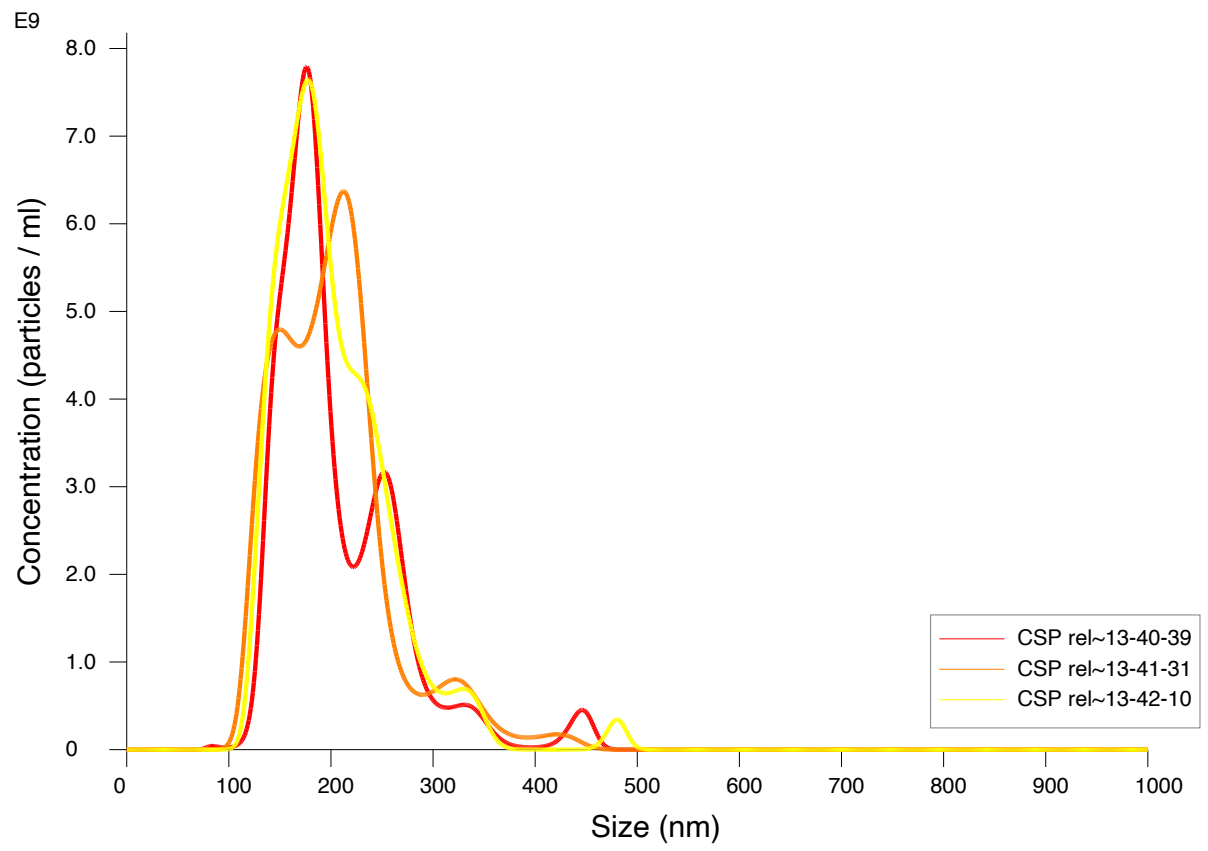

FTLA Concentration / Size graph for Experiment:
CSP released 500X 2025-03-28 13-39-49

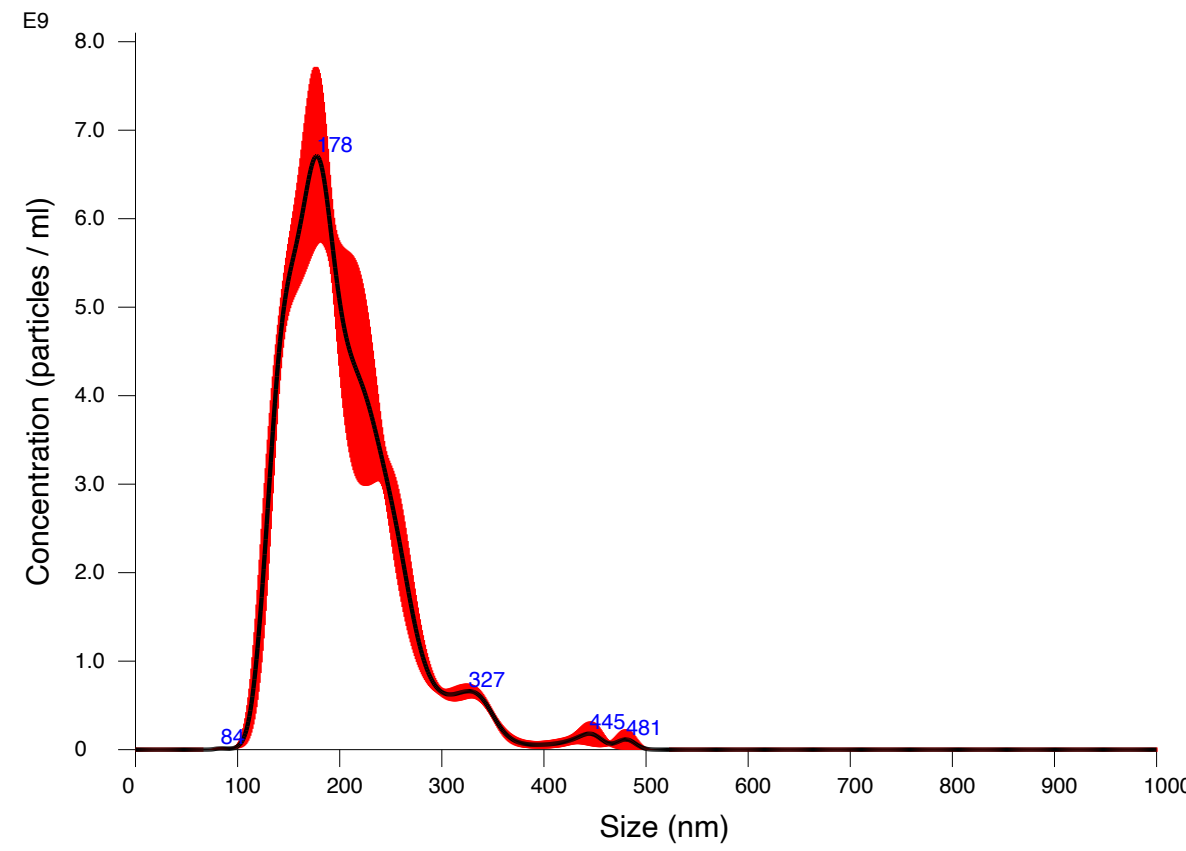

Averaged FTLA Concentration / Size for Experiment:
CSP released 500X 2025-03-28 13-39-49
Error bars indicate + / -1 standard error of the mean

Included Files

CSP released 500X 2025-03-28 13-40-39
CSP released 500X 2025-03-28 13-41-31
CSP released 500X 2025-03-28 13-42-10

Details

NTA Version:	NTA 3.4 Build 3.4.4
Script Used:	SOP Standard Measurement 11-47-07AM 28Mar2025.txt
Time Captured:	13:39:49 28/03/2025
Operator:	Delphine
Pre-treatment:	
Sample Name:	CSP released MVs
Diluent:	1/500
Remarks:	

Capture Settings

Camera Type:	sCMOS
Laser Type:	Green
Camera Level:	15
Slider Shutter:	1206
Slider Gain:	245
FPS	25.0
Number of Frames:	749
Temperature:	23.4 ºC
Viscosity:	(Water) 0.9 cP
Dilution factor:	5 x 10e2
Syringe Pump Speed:	13

Analysis Settings

Detect Threshold:	26
Blur Size:	Auto
Max Jump Distance:	Auto: 10.8 - 11.1 pix

Results

Stats: Merged Data

Mean:	202.9 nm
Mode:	177.5 nm
SD:	58.2 nm
D10:	142.0 nm
D50:	191.1 nm
D90:	272.0 nm

Stats: Mean +/- Standard Error

Mean:	203.0 +/- 1.0 nm
Mode:	188.7 +/- 11.8 nm
SD:	58.3 +/- 1.2 nm
D10:	141.9 +/- 2.8 nm
D50:	191.3 +/- 3.2 nm
D90:	271.7 +/- 2.1 nm
Concentration (Upgrade):	7.37e+11 +/- 4.15e+10 particles/ml
	135.1 +/- 3.6 particles/frame
	127.9 +/- 2.4 centres/frame

Concentration measurements may be unreliable
See summary file for more info

FIGURE S1. NanoSight nanoparticle tracking analysis (NTA) size distribution outputs for stationary phase membrane vesicles. **(A)** Representative NanoSight averaged FTLA concentration/size output for MVs isolated from stationary-phase cultures without CSP induction (MV–NI). **(B)** Representative NanoSight averaged FTLA concentration/size output for MVs isolated from stationary phase cultures following CSP induction (MV–NI). Data represent the average of three technical captures per sample. Mean, mode, standard deviation, and percentile diameters (D10, D50, D90) were calculated using NanoSight NTA software.

TABLE S1. Proteins present at significantly higher levels in MV–CI compared with MV–NI. Proteins are listed with accession numbers, gene identifiers, functional annotations, and $\log_2$ fold-change values (MV–CI vs. MV–NI). Proteins highlighted in grey represent those detected exclusively in MV–CI samples.

Protein accession number	Gene ID	Protein function	Log_2 FC
Q8DWN5	SMU.07	Peptidyl-tRNA hydrolase	6.64
Q8DUC7	SMU.1013c	Mg2+/citrate symporter	6.64
Q8DUC6	SMU.1014	HEAT repeat domain-containing protein	6.64
Q8DUB7	SMU.1025	Transcriptional regulator	6.64
Q8DUB4	SMU.1028	Hydrolase or acyltransferase	6.64
Q8DUB2	SMU.1030	Polyribonucleotide nucleotidyltransferase	6.64
Q8DUA5	SMU.1038c	Response regulator	6.64
Q8DU87	SMU.1057	Acid tolerance operon, SatE	6.64
P96468	SMU.1061	Acid tolerance operon, YlxM	6.64
Q54433	SMU.1075	Probable phosphopantothenoylcysteine decarboxylase	6.64
Q8DU66	SMU.1083c	L-threonylcarbamoyladenylate synthase	6.64
Q8DU56	SMU.1094	ABC transporter, ATP-binding protein	6.64
Q8DU54	SMU.1096	ABC transporter, ATP-binding protein, choline transporter	6.64
Q8DU48	SMU.1105c	Phosphoglycerate mutase-like protein	6.64
Q8DU44	SMU.1109c	Integral membrane protein possible permease	6.64
Q8DU42	SMU.1112c	VOC domain-containing protein	6.64
I6L907	SMU.1113	Sortase	6.64
Q8DU35	SMU.1122	Cytidine deaminase	6.64
Q8DU34	SMU.1123	Deoxyribose-phosphate aldolase	6.64
Q8DWE9	SMU.112c	Transcriptional regulator	6.64
Q8DU23	SMU.1135	Phosphate import ATP-binding protein	6.64
Q8DU21	SMU.1137	Phosphate transport system permease	6.64
Q8DU13	SMU.1146c	Nukacin resistance, response regulator	6.64

Q8DU07	SMU.1152c	DUF1858 domain-containing protein	6.64
Q8DTY2	SMU.1179c	Amino acid ABC transporter, permease protein	6.64
Q02418	SMU.1182	Mannitol-1-phosphate 5-dehydrogenase	6.64
Q8DTW5	SMU.1205c	HNH endonuclease	6.64
Q8DTV8	SMU.1215	Uracil DNA glycosylase	6.64
Q8DTU5	SMU.1228c	Glutamine amidotransferase domain-containing protein	6.64
Q8DTT5	SMU.1238c	Uracil-DNA glycosylase-like domain-containing protein	6.64
Q8DTT0	SMU.1246c	Transcriptional regulator	6.64
Q8DTS0	SMU.1257c	UDP-N-acetylglucosamine kinase	6.64
Q8DTR8	SMU.1259	Site-specific DNA-methyltransferase (adenine-specific)	6.64
Q8DTP9	SMU.1282	Transcriptional regulator	6.64
Q8DTP4	SMU.1289c	Permease, chloride channel	6.64
Q8DTM7	SMU.1306c	Nucleotide-binding protein	6.64
Q8DTM5	SMU.1308	Translation initiation inhibitor	6.64
Q8DWD3	SMU.131	Lipoate--protein ligase	6.64
Q8DTL9	SMU.1314	Esterase	6.64
Q8DTL8	SMU.1315c	ATP-binding protein	6.64
Q8DTK7	SMU.1329c	Transposase	6.64
Q8DTH1	SMU.1372c	Insertion element IS150	6.64
Q8DTG3	SMU.1383	3-isopropylmalate dehydrogenase	6.64
Q8DTF6	SMU.1391c	DUF6287 domain-containing protein	6.64
Q8DTF2	SMU.1395c	Uncharacterized protein	6.64
Q8DTE2	SMU.1406c	NADPH-dependent FMN reductase-like domain-containing protein	6.64
Q8DWC3	SMU.141	DUF1275 domain-containing protein	6.64
Q8DTD6	SMU.1415c	Phosphatases involved in N-acetyl-glucosamine catabolism	6.64
Q8DTD2	SMU.1419	Transcriptional regulator	6.64
Q8DTC2	SMU.1430	Lipid II isoglutaminyl synthase (glutamine-hydrolyzing) subunit	6.64
Q8DTA4	SMU.1454c	Membrane protein	6.64
Q8DTA3	SMU.1456c	Uncharacterized protein	6.64

Q8DT90	SMU.1474c	Ribonuclease Z	6.64
Q8DT89	SMU.1475c	Cystathionine beta-lyase	6.64
Q8DT84	SMU.1482c	AB hydrolase-1 domain-containing protein	6.64
Q8DT80	SMU.1486c	Histidinol-phosphatase	6.64
P26426	SMU.1492	PTS system lactose-specific EIIA component	6.64
P26423	SMU.1496	Galactose-6-phosphate isomerase subunit	6.64
Q8DWB6	SMU.151	Mutacin IV, NlmB	6.64
Q8DT67	SMU.1511c	Acetyltransferase	6.64
Q8DT62	SMU.1520	ABC transporter, glutamine binding protein	6.64
Q8DT56	SMU.1526c	DUF1146 domain-containing protein	6.64
P95785	SMU.1532	ATP synthase	6.64
Q8DT48	SMU.1545c	Citrulline cluster-linked protein	6.64
Q8DT34	SMU.1561	Trk system potassium uptake protein	6.64
Q8DT15	SMU.1582c	GAF domain-containing protein	6.64
Q8DT03	SMU.1598	PTS system, cellobiose-specific IIA component	6.64
Q8DT02	SMU.1599	Transcriptional regulator	6.64
Q8DT01	SMU.1600	PTS system, cellobiose-specific IIB component	6.64
Q8DWA6	SMU.161	Transcriptional regulator	6.64
Q8DSY7	SMU.1619c	Endoribonuclease	6.64
Q8DSY4	SMU.1622	Methionine sulfoxide reductase	6.64
Q8DSW1	SMU.1647c	Transcriptional regulator	6.64
Q8DSS8	SMU.1681c	VOC domain-containing protein	6.64
I6L8Z1	SMU.1693	Magnesium protection factor	6.64
Q8DSS3	SMU.1697c	rRNA methylase	6.64
Q7ZAK8	SMU.1713c	Segregation and condensation protein	6.64
Q8DSP9	SMU.1725	Acylphosphatase	6.64
Q8DW94	SMU.174c	Polymerase nucleotidyl transferase domain-containing protein	6.64
Q8DSL0	SMU.1771c	Polymerase beta nucleotidyltransferase domain-containing protein	6.64
Q8DSK1	SMU.1781	Nucleoside triphosphate/diphosphate phosphatase	6.64

Q8DSH0	SMU.1815	Response regulator	6.64
Q8DSG0	SMU.1827	Biotin transporter	6.64
Q59935	SMU.1839	Mannose-6-phosphate isomerase	6.64
Q07211	SMU.1840	Fructokinase	6.64
Q8DSD1	SMU.1870	DNA mismatch repair protein	6.64
Q8DW83	SMU.187c	tRNA-dihydrouridine synthase	6.64
Q8DSA3	SMU.1905c	Bacteriocin secretion protein	6.64
Q8DS88	SMU.1923c	Transcriptional repressor	6.64
I6L8Z9	SMU.1925c	DUF177 domain-containing protein	6.64
I6L8Y8	SMU.1936c	DNA-directed RNA polymerase subunit delta	6.64
Q93DA2	SMU.1939c	Methionine import ATP-binding protein	6.64
Q8DS75	SMU.1958c	PTS system, mannose-specific IIC component	6.64
Q8DS65	SMU.1970c	Phenylalanyl-tRNA synthetase, beta subunit	6.64
Q8DS53	SMU.1982c	Type II secretory pathway, pseudopilin	6.64
Q8DS42	SMU.1994	ABC transporter, ATP-binding protein	6.64
Q8DS30	SMU.2008	Large ribosomal subunit protein uL30	6.64
Q8DS08	SMU.2043c	D-aminoacyl-tRNA deacylase	6.64
Q8DRZ9	SMU.2054c	DUF3013 domain-containing protein	6.64
Q8DRZ5	SMU.2058	Transcriptional regulator	6.64
Q8DRY6	SMU.2070	UPF0246 protein	6.64
Q8DRY0	SMU.2076c	Transposase	6.64
Q8DRX6	SMU.2080	Bacteriocin transcriptional regulator, BrsR	6.64
Q8DRX1	SMU.2087	3-methyl-adenine DNA glycosylase	6.64
Q8DRW6	SMU.2093	Arginine repressor	6.64
Q8DRW0	SMU.2100c	DUF2179 domain-containing protein	6.64
Q8DRV5	SMU.2106c	Transcriptional regulator	6.64
I6L919	SMU.2109	Multidrug efflux pump	6.64
I6L918	SMU.2111c	Uncharacterized protein	6.64
Q8DRV1	SMU.2113c	Phosphoglycerate mutase	6.64

Q8DRU5	SMU.2119	Osmoprotectant ABC transporter, permease	6.64
Q8DRT5	SMU.2130	Exfoliative toxin	6.64
Q8DRS2	SMU.2147c	LysM domain-containing protein	6.64
Q8DRR4	SMU.2155	Uncharacterized protein	6.64
Q8DW60	SMU.215c	Uncharacterized protein	6.64
Q8DRQ7	SMU.2162c	Ribosomal RNA large subunit methyltransferase	6.64
Q8DW52	SMU.223c	Transposase	6.64
Q8DW50	SMU.225c	Uncharacterized protein	6.64
Q8DWM0	SMU.25	DNA repair protein	6.64
Q8CVC8	SMU.299	Bacteriocin-like peptide, Pep299	6.64
Q8DVZ0	SMU.303	Metallo-beta-lactamase domain-containing protein	6.64
Q8DVY9	SMU.304	tRNA-specific adenosine deaminase	6.64
Q9X670	SMU.307	Glucose-6-phosphate isomerase	6.64
Q8DWL4	SMU.31	Phosphomethylpyrimidine kinase	6.64
Q8DVY1	SMU.328	Carbonic anhydrase	6.64
Q8DVY0	SMU.329	HTH DNA-binding protein	6.64
Q8DWL2	SMU.33	Phage protein	6.64
Q8DVX6	SMU.333	MFS transporter	6.64
Q8DVX3	SMU.337	Membrane protein insertase	6.64
Q8DVX1	SMU.339	DUF998 domain-containing protein	6.64
Q8DVW6	SMU.344	DUF1697 domain-containing protein	6.64
Q8DVW3	SMU.348	Histidine triad (HIT) hydrolase	6.64
Q8DWL0	SMU.35	Phosphoribosylglycinamide formyltransferase	6.64
Q8DVV9	SMU.353	Thiamine diphosphokinase	6.64
Q8DVT3	SMU.384	HTH marR-type domain-containing protein	6.64
Q8DVQ3	SMU.417	Ribosome maturation factor RimP	6.64
Q8DVQ0	SMU.420	Ribosomal protein	6.64
Q8DVP1	SMU.433	Transcriptional regulator	6.64
Q8DVP0	SMU.434	DUF3021 domain-containing protein	6.64

Q8DVN3	SMU.444	Uncharacterized protein	6.64
Q8CWY6	SMU.445	Glycine--tRNA ligase alpha subunit	6.64
Q8DVM8	SMU.451	Uncharacterized protein	6.64
Q8DVL7	SMU.463	Thioredoxin reductase	6.64
Q8DVK0	SMU.485	Membrane protein LiaF	6.64
Q8DVJ6	SMU.490	Pyruvate formate-lyase activating enzyme	6.64
Q8DVJ4	SMU.494	Transaldolase	6.64
Q8DVH9	SMU.509	MmcQ family protein	6.64
Q8DVH8	SMU.510c	Lipoprotein	6.64
Q8DVH4	SMU.515	67 kDa myosin-crossreactive streptococcal antigen	6.64
Q8DVG2	SMU.528c	ABM domain-containing protein	6.64
Q8DVF7	SMU.533	Anthranilate synthase, beta subunit	6.64
Q8DVF4	SMU.536	N-(5'-phosphoribosyl)anthranilate isomerase	6.64
Q8DVF0	SMU.540	Peroxide resistance protein	6.64
Q8DVE2	SMU.549	UDP-N-acetylglucosamine--N-acetylmuramyl-(pentapeptide) pyrophosphoryl-undecaprenol N-acetylglucosamine transferase	6.64
Q8DVD2	SMU.560c	Cytosolic protein	6.64
Q8DVC6	SMU.567	Glutamine ABC transporter, permease component	6.64
Q8DVC0	SMU.573	ADP-dependent (S)-NAD(P)H-hydrate dehydratase	6.64
Q8DVB2	SMU.583	Hemolysin	6.64
Q8DVB1	SMU.584	Arginine repressor	6.64
Q8DVA7	SMU.588	DUF2140 family protein	6.64
Q8DVA4	SMU.592c	Transcriptional regulator	6.64
Q8DV97	SMU.602	Sodium-dependent transporter	6.64
Q8DV83	SMU.622c	HXXEE domain-containing protein	6.64
Q8DV76	SMU.630	Beta-lactamase class A catalytic domain-containing protein	6.64
Q8DV52	SMU.656	Lantibiotic transport system permease, MutE2	6.64
Q8DV47	SMU.661	Transcriptional regulator	6.64
P59311	SMU.663	N-acetyl-gamma-glutamyl-phosphate reductase	6.64

Q8DV36	SMU.680	Gamma-carboxymuconolactone decarboxylase subunit	6.64
Q8DV32	SMU.684	LHH domain-containing protein	6.64
Q8DV19	SMU.700c	Phosphoglycerate mutase-like protein	6.64
Q8DV14	SMU.706c	Proton-coupled thiamine transporter	6.64
Q8DV07	SMU.717	Peptidoglycan branched peptide synthesis protein	6.64
Q8DUZ9	SMU.725c	Calcineurin-like phosphoesterase domain-containing protein	6.64
Q8DUZ1	SMU.737	Serine aminopeptidase S33 domain-containing protein	6.64
Q8DUZ0	SMU.738	Uncharacterized protein	6.64
Q8DUY7	SMU.742	Hydrolase	6.64
Q8DUY6	SMU.743	Hydrolase	6.64
Q8DUY3	SMU.747c	Putative two-component membrane permease complex subunit	6.64
Q8DUY2	SMU.748	Secreted protein	6.64
Q8DUX9	SMU.752	Protein SprT-like	6.64
Q8DUX6	SMU_.58c	DUF3270 family protein	6.64
Q8DUX2	SMU.769	Transcriptional coactivator p15 (PC4) C-terminal domain	6.64
Q8DUW3	SMU.778	Shikimate dehydrogenase	6.64
Q8DUV9	SMU.782	UPF0342 protein	6.64
Q8DUV4	SMU.788	Uncharacterized RNA methyltransferase	6.64
Q8DUU8	SMU.795	Esterase	6.64
Q8DUU7	SMU.796	NmrA-like domain-containing protein	6.64
Q8DUU3	SMU.800	DUF4044 domain-containing protein	6.64
Q8DUT6	SMU.807	Membrane protein	6.64
Q8DUT5	SMU.811	Uncharacterized protein	6.64
Q8DUN5	SMU.869	Ferredoxin reductase	6.64
Q8DUN1	SMU.875c	Transposase, IS150-like	6.64
Q00751	SMU.880	Multiple sugar-binding transport system permease protein MsmG	6.64
Q8DUM3	SMU.896	Uncharacterized protein	6.64
Q8DUM0	SMU.899	DegV domain-containing protein	6.64
Q8DUK3	SMU.924	Thiol peroxidase	6.64

Q8DUJ0	SMU.938	phosphomevalonate kinase	6.64
Q8DWG4	SMU.93c	Transposase	6.64
Q8DUG7	SMU.962	Dehydrogenase	6.64
Q8DUF3	SMU.977	Transcriptional antiterminator	6.64
Q8DUE1	SMU.994	Ribonuclease HII	6.64
Q8DUD6	SMU.999	Uncharacterized protein	6.64
Q8DU57	SMU.1093	ABC transporter, permease protein	6.08
Q8DSR3	SMU.1708	Trk system potassium uptake protein	5.4
Q8DW56	SMU.219	IrrE N-terminal-like domain-containing protein	5.39
Q8DRX4	SMU.2083c	Uncharacterized protein	4.97
Q8DVW0	SMU.352	Ribulose-phosphate 3-epimerase	4.86
Q8DUR4	SMU.839	Folyl-polyglutamate synthetase	4.43
Q8DS50	SMU.1985	ABC transporter ComYB, DNA transport machinery	4.38
Q8DRV4	SMU.2107c	Uncharacterized protein	4.07
Q8DUR7	SMU.836	Peptidase C51 domain-containing protein	3.84
Q8DS97	SMU.1912c	Transposase	3.73
Q8DS73	SMU.1961c	PTS system, sugar-specific enzyme IIA component	3.45
Q8DRU9	SMU.2115	Short-chain dehydrogenase	3.44
Q8DWN7	SMU.05	DUF951 domain-containing protein	3.38
Q8DUD4	SMU.1001	DNA processing Smf protein	3.37
Q8DUV2	SMU.790	HTH cro/C1-type domain-containing protein	3.27
Q8DSD2	SMU.1869	Thioredoxin	3.21
Q8DS57	SMU.1978	Acetate kinase	3.18
Q8DSY6	SMU.1620	PhoH-like protein	3.09
Q8DRS3	SMU.2146c	Transglycosylase SLT domain-containing protein	3.02
Q8DSR1	SMU.1710c	Putative membrane protein insertion efficiency factor	2.91
Q8DTC4	SMU.1428c	Diadenylate cyclase	2.9
Q8DSR5	SMU.1706	DUF4097 domain-containing protein	2.89
Q8DT54	SMU.1537	Glycogen biosynthesis protein GlgD	2.55

Q8DWD2	SMU.132	Hippurate amidohydrolase	2.47
Q8DVT0	SMU.387	tRNA N6-adenosine threonylcarbamoyltransferase	2.39
Q8DT25	SMU.1571	ABC transporter, ATP-binding protein, MsmK-like protein	2.36
Q8DTQ5	SMU.1272	ATP phosphoribosyltransferase regulatory subunit	2.33
Q8DU40	SMU.1116c	DUF4391 domain-containing protein	2.19
Q8DU25	SMU.1133	Phosphate-specific transport system accessory protein PhoU	2.19
Q8DTB8	SMU.1436c	Uncharacterized protein	2.19
Q8DV17	SMU.702c	Cys-tRNA(Pro)/Cys-tRNA(Cys) deacylase	2.14
Q8DV48	SMU.660	Histidine kinase	2.09

TABLE S2. Proteins present at significantly lower levels in MV–CI compared with MV–NI. Proteins are listed with accession numbers, gene identifiers, functional annotations, and $\log_2$ fold-change values (MV–CI vs. MV–NI). Proteins highlighted in grey represent those detected exclusively in MV–NI samples.

Protein accession number	Gene ID	Protein function	$\log_2$ FC
Q8DWC6	SMU.138	Malate permease	−5.05
Q8DUD5	SMU.1000	Uncharacterized protein	−6.64
Q8DUB1	SMU.1031	Excisionase	−6.64
Q8DU27	SMU.1131c	Lipoprotein	−6.64
Q8DTX0	SMU.1197	TVP38/TMEM64 family membrane protein	−6.64
Q8DTN6	SMU.1297	Probable bifunctional oligoribonuclease	−6.64
Q8DTI3	SMU.1356c	Transposase	−6.64
Q8DTH4	SMU.1368	Uncharacterized protein	−6.64
Q8DTB6	SMU.1438c	Zn-dependent protease	−6.64
Q8DTA2	SMU.1459c	Uncharacterized protein	−6.64
Q8DSV4	SMU.1655c	Uncharacterized protein	−6.64
Q8DSS1	SMU.1700c	Uncharacterized protein	−6.64
Q8DSR9	SMU.1702c	Phosphatase	−6.64
Q8DSK9	SMU.1772c	Uncharacterized protein	−6.64
Q8DSA0	SMU.1908c	Uncharacterized protein	−6.64
I6L8Y2	SMU.1927	ABC transporter, ATP-binding protein	−6.64
Q8DW79	SMU.195c	Phage protein	−6.64
Q8DS21	SMU.2019	Large ribosomal subunit protein uL29	−6.64
Q8DRU3	SMU.2121c	YCII-related domain-containing protein	−6.64
Q8DW40	SMU.236c	Transcriptional regulator	−6.64
I6L915	SMU.312	PTS system, sorbitol phosphotransferase enzyme IIBC	−6.64
Q8DVW4	SMU.346	NADH dehydrogenase NAD(P)H nitroreductase	−6.64
Q8DVQ1	SMU.419	YlxR domain-containing protein	−6.64

Q8DVP4	SMU.429c	YgjV family protein	−6.64
Q8DVK5	SMU.479	DNA-directed RNA polymerase subunit omega	−6.64
Q8DVI7	SMU.501	Uncharacterized protein	−6.64
Q8DVI5	SMU.503c	Lipoprotein	−6.64
Q8DVH2	SMU.517	Phosphopantetheine adenylyltransferase	−6.64
Q8DVE6	SMU.545	Transposase	−6.64
Q8DVA8	SMU.587	SGNH hydrolase-type esterase domain-containing protein	−6.64
Q8DV69	SMU.637c	Uncharacterized protein	−6.64
Q8DV68	SMU.638	16S pseudouridylate synthase	−6.64
Q8DV18	SMU.701c	Inner membrane component domain-containing protein	−6.64
Q8DUX7	SMU.757	Gas vesicle protein	−6.64
Q8DUL1	SMU.914c	DUF3021 domain-containing protein	−6.64
Q8DUJ8	SMU.929c	Integral membrane protein	−6.64
Q8DUG3	SMU.967	Tetrahydrofolate synthase	−6.64
